# Effect of Danlu capsules for the treatment of breast hyperplasia with mastalgia: a multicenter, double-blind, randomized controlled trial protocol

**DOI:** 10.3389/fmed.2025.1687673

**Published:** 2025-10-21

**Authors:** Qianqian Guo, Ziqian Luo, Jihan Huang, Rui Xu, Yan Dai, Qianjun Chen

**Affiliations:** ^1^State Key Laboratory of Traditional Chinese Medicine Syndrome/Breast Disease Specialist Hospital, The Second Affiliated Hospital of Guangzhou University of Chinese Medicine, Guangdong Provincial Hospital of Chinese Medicine, Guangdong Provincial Academy of Chinese Medical Sciences, Guangzhou, Guangdong, China; ^2^Guangdong Provincial Key Laboratory of Clinical Research on Traditional Chinese Medicine Syndrome, Guangdong Provincial Hospital of Chinese Medicine, Guangzhou, Guangdong, China; ^3^Postdoctoral Research Center, Guangdong Provincial Hospital of Chinese Medicine, Guangzhou, Guangdong, China; ^4^The Second Clinical College of Guangzhou University of Chinese Medicine, Guangzhou, Guangdong, China; ^5^Center for Drug Clinical Research, Shanghai University of Traditional Chinese Medicine, Shanghai, China; ^6^Chinese Medicine Guangdong Laboratory, Guangzhou, Guangdong, China

**Keywords:** Danlu capsules, breast hyperplasia, mastalgia, randomized controlled trial, protocol

## Abstract

**Background:**

Mastalgia is a prevalent condition among women. Conservative treatment is initially recommended, and tamoxifen is considered a second-line option, but it can cause menopausal-like symptoms. Chinese patent medications are proposed as alternatives, yet evidence remains insufficient. Danlu capsules, which are recommended in certain clinical practice guidelines for breast hyperplasia in China, have demonstrated therapeutic effects on breast pain. However, high-quality randomized controlled trials are lacking to clarify Danlu capsules’ efficacy in treating breast pain. This study aims to examine the effectiveness and safety of Danlu capsules for the treatment of mastalgia.

**Methods:**

This study is a multicenter, randomized, double-blind, placebo-controlled, and superiority clinical trial involving 264 participants, which will be randomly assigned in a 1:1 ratio to either receive Danlu capsule or placebo via a centralized system. All participants will undergo an eight-week treatment period and an additional 4 weeks of follow-up. The primary outcome will be the breast pain assessed using the visual analog scale (VAS). Secondary outcomes will include the evaluation of the quality-of-life questionnaire, the self-rating anxiety questionnaire, and the self-rating depression questionnaire. Safety monitoring and adverse event recording will be implemented throughout the study. Efficacy will be assessed through covariance analysis for primary endpoints and t-tests for secondary outcomes, with mixed models for repeated measures comparing breast pain VAS score changes at weeks 4, 8, and 12. All statistical analyses will be conducted using SAS (9.4), and no interim analyses will be performed.

**Discussion:**

This trial will evaluate the clinical efficacy and safety of Danlu capsules in treating breast hyperplasia with mastalgia. The findings are expected to provide an evidence-based treatment option supporting the potential role of Chinese patent medicine in the management of breast pain.

**Clinical trial registration:**

http://itmctr.ccebtcm.org.cn/, ITMCTR2025000715.

## 1 Background

Breast hyperplasia is a relatively common disease among women, which is primarily defined by cyclical breast pain and the presence of multiple breast lumps. Breast pain, also known as mastalgia, is the main reason why women visit the gynecological clinic for breast-related problems (1). Previous reviews have reported that breast pain is a prevalent condition that affects as many as 70–80% of women over the course of their lives (2, 3). A research study involving a questionnaire survey of 1,214 women, with an average age of 22.87, revealed that 35.5% of the participants reported breast pain (4). Further, research by Brown et al. (5) has indicated that breast pain complaints are more common among premenopausal women than among postmenopausal women.

Patients with breast pain have two major concerns: the severity of pain that impairs their quality of life, and the fear of breast cancer (6). Although the pain is typically mild and self-limiting, approximately 15% of the women affected seek medical treatment (7). Currently, a conservative approach is typically advocated as the initial treatment for managing breast pain, encompassing physical support measures such as wearing supportive garments (8–10), applying warm or cold compresses, or engaging in gentle massage techniques. Additionally, over-the-counter pain relief medications, such as acetaminophen and nonsteroidal anti-inflammatory drugs, are often recommended (11). In cases where breast pain continues to be a concern after 6 months of conservative treatment, tamoxifen is often considered a second-line therapy (12). This treatment is typically administered for 1 to 3 months, either until the pain is eased or until side effects necessitate discontinuation. It is crucial to be aware that tamoxifen can lead to menopausal-like symptoms, such as hot flashes and vaginal discharge (13).

The Clinical Application Guidelines for the Treatment of Breast Hyperplasia with Chinese Patent Medicine (14) suggest the use of traditional Chinese medicines, including Ruyi sanjie capsules, Danlu capsules, Honghua xiaoyao tablets, and Xiaoyao pills, for the management of breast pain. However, it should be highlighted that the current evidence supporting the efficacy of these treatments is of a lower grade, mainly classified as Grading of Recommendations, Assessment, Development and Evaluation (GRADE) 1C or 2C. Therefore, there is a need for more robust evidence to support these treatment options, which is where the significance of conducting high-quality randomized controlled trials comes into play.

Danlu capsules are composed of eight herbs, including Ostrea gigas Thunberg, *Polygonum multiflorum* Thunb., etc. This capsule has been listed as a recommended medication in several clinical guidelines and consensuses, and has undergone many clinical studies (15, 16). For example, a Phase IV clinical study involved 2,294 patients (17), showing a marked and statistically significant decrease in breast pain intensity after one and two menstrual cycles. However, as a single-arm trial without a control group, this study still provides insufficient evidence to support the clinical application of Danlu capsules for breast pain improvement.

This project focuses on the prominent issue of the lack of a standard, safe treatment for mastalgia, with the core goal of relieving breast pain symptoms in clinical settings. Since no accepted standard-of-care intervention exists for mastalgia, the placebo comparator is designed in this context. Therefore, a prospective, multicenter, randomized, double-blind, placebo-controlled, parallel-group trial with a 1:1 allocation ratio will be conducted under a superiority framework to clarify the clinical efficacy of Danlu capsules in treating breast hyperplasia accompanied by breast pain, thereby offering an effective and safe clinical treatment plan for patients with breast hyperplasia accompanied by breast pain.

## 2 Methods

### 2.1 Study design

**Figure 1:**
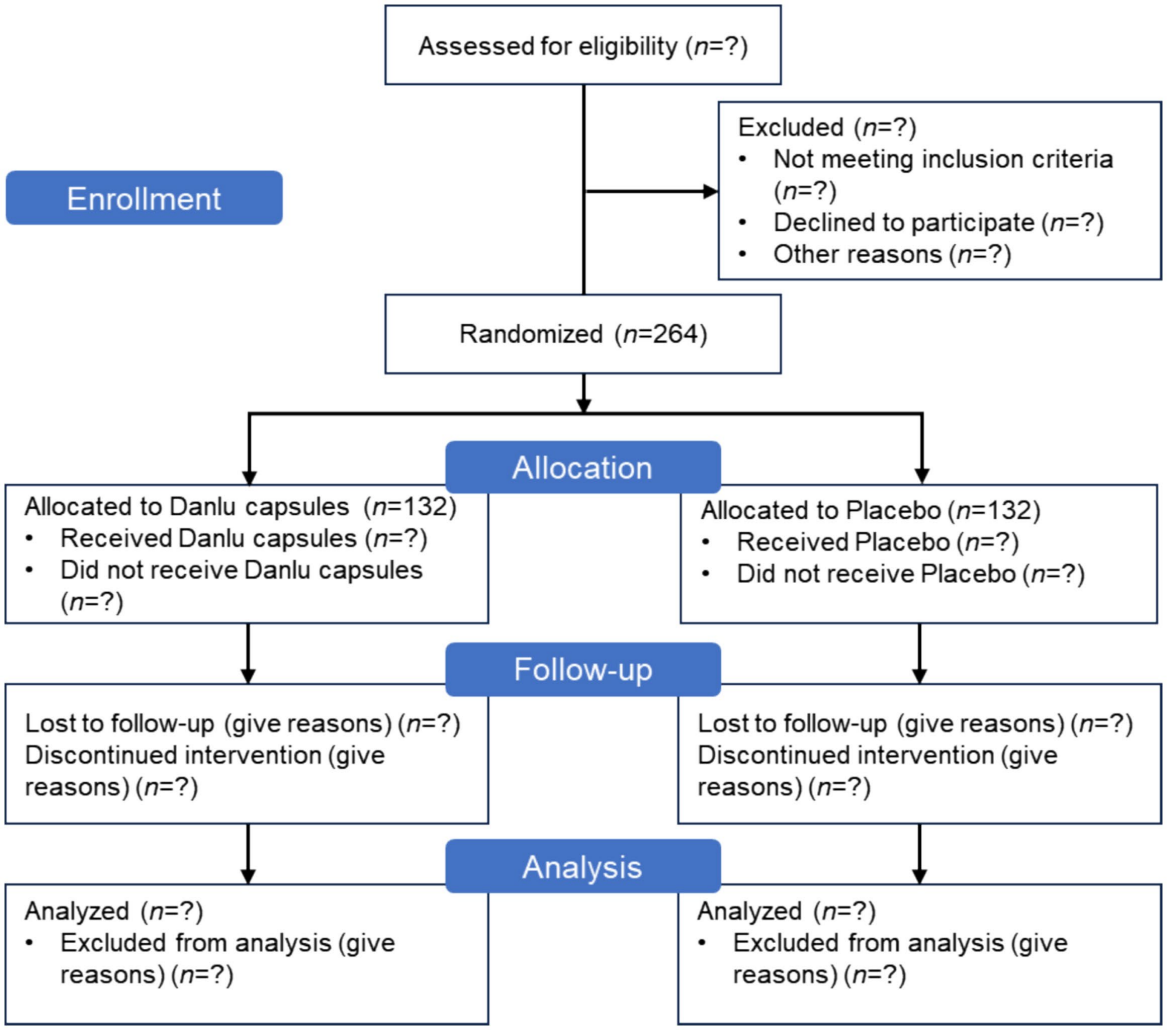
CONSORT flow diagram for Danlu capsules clinical trial.

This is a prospective, randomized, double-blind, placebo-controlled, multicenter clinical trial designed to evaluate the efficacy and safety of Danlu capsules in relieving breast pain. Written informed consent will be obtained from all participants prior to enrollment. Patients will be randomly assigned to either the treatment group, which will receive Danlu capsules, or the placebo group, in a 1:1 ratio. The flow of participants through the study is concisely illustrated in Figure 1. This protocol is reported in accordance with the Standard Protocol Items: Recommendations for Interventional Trials (SPIRIT) 2025 guidelines (18), with the checklist available in Supplementary file 1.

### 2.2 Patient selection

Patients diagnosed with breast hyperplasia (19) and experiencing breast pain who present at the outpatient clinics of the Guangdong Provincial Hospital of Chinese Medicine and our collaborating centers (Table 1) are eligible for inclusion in the study. The diagnostic criteria for breast hyperplasia are delineated as follows: (1) Breast pain: there are varying degrees of distending pain, stabbing pain, or dull pain in the breasts, which can be bilateral or unilateral and change with the menstrual cycle or emotional fluctuations; (2) Breast lumps: one or both breasts may have single or multiple lumps of unequal size, irregular shape, firm texture, and unclear boundaries, with tenderness upon palpation; (3) Ancillary examinations: ultrasound—shows disordered or unevenly enhanced echoes in the glandular tissue, with visible cystic dilation of the ducts; mammography—shows a relatively uniform density increase in the breast shadows, which can appear in one or multiple quadrants; pathology—shows varying degrees of increase and dilation in breast ducts and acini, with hyperplasia of interstitial fibrous tissue. A definite diagnosis can be made if both (1) and (2) are present, along with any one of the items in (3).

**Table 1:** The hospitals participating in this study.

Code	Participating hospitals
01	Guangdong Provincial Hospital of Chinese Medicine
02	Yueyang Hospital of Integrated Traditional Chinese and Western Medicine, Shanghai University of Traditional Chinese Medicine
03	Sanya Maternal and Child Health Hospital
04	Tongzhou Maternal & Child Health Hospital of Beijing
05	Luoyang Dongfang People’s Hospital
06	Sanya Central Hospital (Hainan Third People’s Hospital)
07	Beijing Shijitan Hospital, Capital Medical University
08	Zhengzhou Traditional Chinese Medicine Hospital
09	Sichuan Second Hospital of Traditional Chinese Medicine
10	Affiliated Hospital of Hebei University of Engineering

Study recruitment advertisements will be displayed on notice boards at the participating hospitals. These advertisements provide a concise overview of the participant criteria, the medications involved, the medical examinations required, and the methods for enrolling in the study. For those deemed ineligible or who choose not to participate, we will document their basic demographic information and the reasons for their non-participation. Patients will be assessed before treatment and at 4 and 8 weeks of treatment. After completing the eight-week treatment course, a follow-up assessment will be conducted 1 month after stopping the medication. The specific assessments at each visit are detailed in Table 2.

**Table 2:** The schedule of enrolment, interventions, and assessments.

Item	Study period				
	Enrolment		Follow-up		
Timepoint	Screening stage	Visit 0 (Day 0)	Visit 1 (Week 4 ± 4d)	Visit 2 (Week 8 ± 4d)	Visit 3*
Enrolment					
Eligibility screen	X				
Informed consent	X				
Demographic information	X				
Interventions					
Danlu capsules		X	X	X	
Placebo		X	X	X	
Efficacy assessment					
Breast pain assessment		X	X	X	X
Breast lump assessment		X		X	X
Quality of Life Scale (SF-36)		X	X	X	X
Anxiety and depression assessment scale		X	X	X	X
Hormones (estrogen, progesterone, prolactin)		X		X	
Interleukin-6		X		X	
Tumor necrosis factor-alpha		X		X	
Safety assessment					
Vital sign	X	X	X	X	X
Urinary pregnancy test	X				
Blood routine		X		X	
Serum biochemicals		X		X	
Adverse events recorded			X	X	X
Others					
Issue medications		X	X		
Recycle medications			X	X	
Combination therapy	X	X	X	X	X
eCRF	X	X	X	X	X

*One month after discontinuing the medication±10d. SF-36, 36-Item Short-Form; CRF, Case report form.

### 2.3 Inclusion and exclusion criteria

The inclusion criteria were as follows: (1) female patients aged 18 to 65 years (inclusive of 18 and 65 years); (2) meeting the diagnostic criteria for breast hyperplasia and having a visual analog scale (VAS) score for breast pain of ≥4 points; (3) voluntarily participating in the study, signing an informed consent form (see Supplementary file 2), demonstrating good compliance, and being willing to cooperate with follow-up visits. The exclusion criteria were as follows: (1) patients with other breast-related diseases, such as acute mastitis, benign or malignant breast tumors; (2) patients with other diseases causing chest pain; (3) patients with severe primary diseases of the liver, kidneys, or hematopoietic system; (4) patients with biochemical indicators showing total bilirubin (TBIL) > 1.5 × upper limit of normal (ULN), alanine aminotransferase (ALT) > 1.5 × ULN, or aspartate aminotransferase (AST) > 1.5 × ULN; (5) pregnant or lactating women; (6) patients with an allergic constitution or allergies to the ingredients of the study medication; (7) patients with a confirmed diagnosis of anxiety disorder; (8) patients who had participated in other clinical trials within the past 3 months.

To ensure the external validity and generalizability of the study findings, the inclusion and exclusion criteria have been designed to reflect the real-world clinical context of patients with breast pain. In the clinical setting, the majority of patients are either treatment-naïve or have exclusively used traditional Chinese medicine and Chinese patent medications, with minimal reliance on tamoxifen or conventional analgesics for the treatment of breast pain. Given this context, enrollment has been not restricted based on prior use of Danlu capsules or other medications for breast pain. However, to mitigate potential biases arising from prior medication use, data will be subjected to stratified analyses based on whether other medications were used within the past month when necessary. This approach will help ensure that the results are robust and reliable.

### 2.4 Dropout and study termination criteria

After randomization, any patient who withdraws from the study at any time during the study period, regardless of the reason, will be considered a dropout case. For patients who decide to withdraw, a final examination should be conducted (if possible), and safety and efficacy data should be collected for further analysis. The researcher should contact the patients or their responsible family members to confirm the reason for withdrawal and recover any remaining medication. If a patient withdraws due to adverse events, these events should be recorded in the case report form (CRF). Reasons for dropout include violation of the trial protocol (poor compliance), loss to follow-up (patient withdraws without permission), researcher-initiated termination, and intolerable adverse events.

To protect the rights and interests of the participants, ensure the quality of the trial, and avoid unnecessary economic losses, researchers should carefully document the reasons for terminating the trial. The study may be terminated under the following circumstances: (1) the researcher deems it medically necessary for the participant to withdraw from the trial; (2) the participant requests to stop the trial; (3) the participant experiences a severe allergic reaction related to the study medication; (4) the participant experiences adverse symptoms, signs, or abnormal examination results related to the study medication, and the researcher determines that terminating the study is necessary after careful evaluation; (5) the participant becomes pregnant during the study period.

### 2.5 Intervention

Eligible patients will be randomly assigned to one of two groups:

1. Treatment group: Danlu capsules, 2.0 g (four capsules) per dose, three times daily, for 8 weeks.
2. Placebo group: placebo, 2.0 g (four capsules) per dose, three times daily, for 8 weeks.

Danlu capsules and placebo will be supplied by Suzhou Pharmaceutical Group Co., Ltd. (national drug approval number Z20150004). The placebo capsules must be indistinguishable from Danlu capsules in appearance, color, taste, packaging, and administration method. The placebo is primarily composed of soluble starch and heavy calcium carbonate, which account for 56.84 and 29.69% of its total composition, respectively. To closely replicate the aroma of Danlu capsules, a trace amount of paeonol has been incorporated. Further, minute quantities of flavoring agents and food coloring have been introduced to ensure that the taste and appearance of the placebo closely resemble those of Danlu capsules. Study medications must be stored securely in a dry place to maintain their integrity. Each clinical trial site will designate a responsible individual to oversee the retrieval and management of study medications.

Danlu capsules, which have been on the market for many years, consist of eight medicinal herbs as detailed in Table 3. Research has suggested that Kun Bu (Laminaria japonica Aresch.) may be involved in biological processes such as signal transduction, apoptosis, cell proliferation, and inflammatory responses (20). Furthermore, He Shou Wu (*Polygonum multiflorum* Thunb.) has been recognized for its definitive lipid-lowering effect, potentially involving the regulation of the PI3K-Akt, HIF-1, and estrogen signaling pathways (21).

**Table 3:** Components and dose of Danlu capsules.

Chinese name	English name	Weight (%)*
Mu Li	Ostrea gigas Thunberg	22.22%
He Shou Wu	*Polygonum multiflorum* Thunb.	11.11%
She Chuang Zi	*Cnidium monnieri* (L.) Cuss	11.11%
Lu Jiao	*Cervus elaphus* Linnaeus	11.11%
Mu Dan Pi	*Paeonia suffruticosa* Andr.	11.11%
Chi Shao	*Paeonia lactiflora*	11.11%
Yu Jin	Curcuma wenyujin Y. H. Chen et C. Ling	11.11%
Kun Bu	Laminaria japonica Aresch.	11.11%

*The weight of every single herb in each bag of Danlu capsules (0.5 g).

During the treatment period, the use of Western pharmaceuticals, traditional Chinese medicines, Chinese patent medicines, or external therapies that might duplicate the effects or target the same conditions as the study medication is prohibited. If there is a need to use other permissible medicines, it is essential to meticulously document their names, the justification for their use, the dosage administered, and the treatment duration on the case observation form. This detailed record-keeping is crucial for maintaining the study’s integrity and ensuring the accuracy of the research outcomes.

### 2.6 Randomization and blinding

This trial employs a centralized randomization method. Random numbers and corresponding groups are generated using SAS 9.4. The randomization table (blind code) will be released to the statistician by the randomization coder, with sponsor approval, after study completion, finalization of the statistical analysis and data audit report, and database lock. The trial follows a double-blind design. Drug blinding is conducted by randomization unit personnel and individuals unrelated to the trial. Drug packaging numbers and verification codes, generated by SAS, are labeled on the drug tags.

After data entry into the electronic data capture (EDC) system, the database will be locked following query, verification, and blind audit. The principal investigator and statistician will then unblind the data to identify subject groups and interventions and conduct statistical analysis. In the event of an emergency, such as a serious adverse event or complication, emergency unblinding can be performed with the principal investigator’s confirmation and signature. The sponsor and principal investigator must be notified within 24 h, with reasons for unblinding provided.

### 2.7 Sample size calculation

Given that no placebo-controlled study had been conducted for Danlu capsules prior to this study, the sample size estimation was based on the available data. According to the literature (17, 22), the VAS score for breast pain decreases by 3.2 points after two menstrual cycles of treatment with Danlu capsules (17), while the score in the control group (placebo) decreases by approximately 1.5 points (22), with a standard deviation of 3.4 points. The sample size for this superiority trial was calculated using a t-test. Based on a superiority margin of 0, a two-sided *α* of 0.05, and *β* of 0.05 (power = 95%) with 1:1 allocation, the required sample size per group was determined to be 105 using PASS 21.0 software. Considering the dropout rate, the sample size was increased by 20%, resulting in 132 cases for each group, making a total of 264 cases.

### 2.8 Data collection

#### 2.8.1 Primary and secondary outcomes

Breast pain is assessed using the VAS before and after treatment, with 0 points indicating no pain and 10 points representing the most severe pain. Higher scores reflect greater pain intensity. The primary outcome is defined as the change in the breast pain VAS score from baseline to the 8-week time point.

In prior research on oral medications for breast hyperplasia, the numerical rating scale score for the most painful day in the placebo group was found to decrease by 1.52 points after the second menstrual cycle (22). According to the phase IV study of Danlu capsules (17), the difference in VAS scores for breast pain between before and after two menstrual cycles of treatment with Danlu capsules was 3.29 points. Therefore, the minimal clinically important difference (MCID) in VAS scores for breast pain between the Danlu capsules treatment group and the placebo group was set at 1.7 points (3.29 minus 1.52). This implies that only a statistically significant difference between the Danlu capsules group and the placebo group, with an intergroup difference greater than 1.7 points, is deemed to have clinical significance.

Secondary outcomes to be assessed are as follows:

1. 36-Item Short-Form: This questionnaire comprises 36 items that evaluate eight dimensions of health-related quality of life, including physical function, physical role, bodily pain, general health, vitality, social function, emotional role, and mental health. Participants will complete this assessment at the start of the study and post-treatment.
2. Anxiety and depression assessment scales: Patients will undergo assessments using the patient health questionnaire-9 (PHQ-9), generalized anxiety disorder-7 (GAD-7), self-rating depression scale (SDS), and self-rating anxiety scale (SAS) at baseline and following drug intervention. The PHQ-9 is a screening tool for depressive symptoms, consisting of 9 items that reflect the respondent’s experiences over the past 2 weeks. The GAD-7 is a brief questionnaire designed to measure symptoms of generalized anxiety disorder over the same period, with seven items that assess various psychological and physiological aspects of anxiety. The SDS is a widely recognized self-report measure for depressive symptoms, encompassing 20 items that cover a range of depressive manifestations such as low mood, loss of interest, and reduced energy. The SAS is a self-report instrument for assessing anxiety levels, including 20 items that address various facets of anxiety, such as tension, palpitations, and fear.
3. Changes in breast lumps: Skilled medical professionals will utilize breast ultrasound and other diagnostic methods to measure the size, number, and texture of breast lumps, tracking changes in these characteristics before and after treatment.
4. Hormone regulation indicators: The study will monitor changes in serum levels of estrogen, progesterone, and prolactin before and after treatment.
5. Exploration of drug mechanism of action: The study will also investigate changes in biomarkers such as interleukin-6 (IL-6) and tumor necrosis factor-alpha (TNF-*α*) in patients’ serum before and after treatment.

#### 2.8.2 Safety outcomes

During the treatment period, vital signs—including heart rate, respiration, and blood pressure—will be monitored and evaluated both before and after treatment. Additionally, adverse reactions potentially related to the medication, such as nausea, vomiting, gastric discomfort, hot flashes, and night sweats, will also be observed. Blood tests, including complete blood count, liver and kidney function, and coagulation function, will be conducted before and after treatment. Any adverse reactions occurring during the study will be recorded, and their severity and potential causal relationship with Danlu capsules will be assessed.

Adverse events are defined as any adverse medical events that occur in patients after medication administration, which may or may not be related to the treatment. All adverse events that occur during the trial process, including laboratory test abnormalities, must be carefully inquired about and investigated. All adverse events must be assessed for their nature, severity, and relevance to the medication and strictly recorded in the CRF. Causality assessment between adverse events and the investigational product will be performed in strict accordance with the five-category classification (definitely related, probably related, possibly related, probably unrelated, and unrelated) as specified in the *Technical Guidelines for Evaluation of the Correlation of Adverse Events in Drug Clinical Trials (Trial Implementation)* issued by the Center for Drug Evaluation (CDE) of the National Medical Products Administration (NMPA) in 2024 (23). During this clinical study, if any harm or serious adverse events related to the trial occur, medical treatment will be provided by the participant’s physician and the hospital.

### 2.9 Data management

The data manager will design a CRF in accordance with the protocol, incorporating logical checks from the data verification plan. Data from original records will be entered into the EDC system by trained and qualified investigators. Queries identified through EDC system checks and manual reviews by monitors and data managers will be promptly addressed by the investigators. After data entry and source data verification are completed, investigators will electronically sign off on the data. Any revisions made after the electronic signature will require a new signature. Upon mutual approval by key stakeholders—the principal investigator, sponsor, statistical analyst, and data manager—the database will be locked by the data manager. The data manager will then submit the database to the statistician. After the completion of the statistical analysis, the data manager will close the EDC, marking the end of the data management phase. In this study, the decision was made not to establish a data monitoring committee. This choice aligns with the study design, which does not incorporate interim analyses.

### 2.10 Quality assurance and quality control

Detailed standard operating procedures (SOPs) will be established before the project commences. These SOPs will provide detailed operational guidelines for each step of the process and clearly define the responsibilities of all project participants. Following the establishment of these SOPs, specialized training sessions will be conducted. This training is mandatory for everyone involved in the project, including researchers, monitors, data managers, and drug administrators, to ensure proficiency in their respective SOPs. The responsible unit will regularly conduct rigorous project audits according to the SOPs. Any identified issues will be addressed promptly. In cases where problems are significant, it may be necessary to revoke the research privileges of the individual researcher or even the entire research unit to maintain the integrity and quality of the project.

### 2.11 Statistical analysis

The full analysis set (FAS) and per-protocol set (PPS) will be used for baseline and efficacy analyses, while the safety set (SS) will be used for the safety analysis. For missing values of primary outcomes in efficacy analyses: the last observation carried forward method will be adopted for imputation in the primary analysis, and multiple imputation will be used for the missing primary outcome data as a sensitivity analysis to verify result robustness. We will calculate the proportions of randomized participants who completed the trial or withdrew prematurely, and record the reasons for these decisions. Further, we will categorize and analyze the reasons for exclusion from the PPS, and provide a detailed examination of demographic and baseline characteristic analyses. This will include descriptive statistics for continuous variables (mean, standard deviation, range) and categorical data (frequency, proportion).

Medication adherence will be evaluated, focusing on participants whose intake falls within the 80–120% range, using the chi-square test or Fisher’s exact test. The exposure to medication and differences between groups will be assessed using t-tests. Concomitant medication use will be categorized, analyzed, and compared between groups using the same statistical methods. Efficacy will be determined through analyses of primary and secondary endpoints: covariance analysis will be used for primary endpoints to compare the breast pain VAS score at week 8 with the baseline (with treatment group and study center as fixed effects and baseline value as a covariate), and t-tests will be used for secondary outcomes (incorporating data from both the PPS and FAS). The changes in breast pain VAS scores from baseline at week 4, week 8, and week 12 (1 month after discontinuation of medication) will be compared between groups using the mixed model for repeated measures. The model will include treatment group, time, and the interaction term of group multiply by time as fixed effects, with baseline value as a covariate. Statistical analysis will be performed using SAS software (version 9.4), with a *p*-value ≤ 0.05 indicating significance, as outlined in the statistical analysis plan. The study will not include interim analyses. The details of the statistical analysis plan are provided in Supplementary file 3.

### 2.12 Dissemination

No data will be disclosed to any third party in any form without the prior written consent of the sponsor. All data and analyses will remain blinded until the study results are published. The final results will be published in relevant academic journals as research articles.

## 3 Discussion

Mastalgia is a common condition among women and can be categorized into cyclical mastalgia, noncyclical mastalgia, and extra-mammary or chest wall pain (24). Breast pain is generally a self-limiting condition that resolves on its own and does not pose an immediate threat (25). For most patients with cyclical mastalgia who have no underlying cause, reassurance is the mainstay of treatment (26). However, it is essential to monitor the condition, as persistent breast pain may indicate the need for more specialized care. Patients are advised to consult healthcare professionals to explore appropriate treatment options. Currently, Western medical approaches to breast hyperplasia have certain limitations, which underscores the necessity for further investigation to develop more effective therapies.

Patients sometimes consider complementary and alternative medicine to manage mastalgia. A previous systematic review demonstrated that herbs are effective in reducing the severity of cyclic mastalgia (27, 28). Evening primrose oil and vitamin E have been reported to reduce breast pain severity, offering an option for patients (29–31). Additionally, complementary and alternative therapies for mastalgia also include internal administration of herbal formulas. Some studies have shown the benefits of herbal formulas for patients with breast pain (32–34), although high-quality randomized controlled trials are limited. The Clinical Application Guidelines for the Treatment of Breast Hyperplasia with Chinese Patent Medicine (14) suggest that various Chinese patent medicines should be used based on Chinese medicine syndrome differentiation.

In terms of risk factors, a cross-sectional study involving 415 Turkish women identified several factors associated with mastalgia, including intense stress, daily consumption of coffee and chocolate, a history of breast surgery, and the menstrual patterns (35). Research also indicated that premenopausal women with breast pain had significantly higher levels of anxiety and lower quality of life compared to healthy controls (36), while another study found that anxiety and depression were more prevalent among mastalgia patients (37). Further, a study has shown that breast pain during the luteal phase of the menstrual cycle may be related to a higher serum estrogen-to-progesterone ratio (38). Therefore, quality-of-life questionnaire, anxiety and depression assessment scales, and hormone indicators will be collected at both baseline and the end of study.

In 2003, Ramakrishnan et al. investigated the relationship between mastalgia and the expression of inflammatory cytokines, including IL-6, interleukin-1β (IL-1β), and TNF-*α*. They compared tissue samples from 29 premenopausal women with mastalgia and 29 age-matched women without pain. The study found that the expression levels of TNF-α and IL-6 tended to be slightly lower in patients with pain. However, limited studies have clarified the relationship between inflammation and mastalgia. As such, blood samples will be collected to explore the relationship between Danlu capsules and these two inflammatory cytokines.

This study was meticulously designed to adhere to a consistent methodology, guided by experts in statistics and methodologists. It involved a comprehensive selection of multiple research centers and emphasized the importance of maintaining high-quality data. By selectively choosing the centers and closely monitoring the data, we aimed to ensure that the results were accurate and reliable. This commitment to detail underscores our dedication to conducting a study that meets the rigorous standards expected in scientific research.

## Glossary

TCM: Traditional Chinese medicine
GRADE: Grading of Recommendations, Assessment, Development and Evaluation
SPIRIT: Standard Protocol Items: Recommendations for Interventional Trials
SF-36: 36-Item Short-Form
VAS: Visual analog scale
TBIL: Total bilirubin
ULN: Upper limit of normal
ALT: Alanine aminotransferase
AST: Aspartate aminotransferase
CRF: Case report form
EDC: Electronic data capture
MCID: Minimal clinically important difference
PHQ-9: Patient health questionnaire-9
GAD-7: Generalized anxiety disorder-7
SDS: Self-rating depression scale
SAS: Self-rating anxiety scale
IL-6: Interleukin-6
TNF-*α*: Tumor necrosis factor-alpha
CDE: Center for Drug Evaluation
NMPA: National Medical Products Administration
SOPs: Standard operating procedures
FAS: Full analysis set
PPS: Per-protocol set
SS: Safety set
