# Supplementary material for: Effect of Danlu capsules for the treatment of breast hyperplasia with mastalgia: a multicenter, double-blind, randomized controlled trial protocol
Source: Front Med (Lausanne). 2025 Oct 21;12:1687673. doi: 10.3389/fmed.2025.1687673 (PMC12582957; doi:10.3389/fmed.2025.1687673)
Supplement: Supplementary file 2 [file Data_Sheet_2.pdf]

Prospective, Randomized, Double-Blind, Multicenter Clinical Study on Danlu Capsule for the Treatment of Mammary Gland Hyperplasia

Dear Patient:

We sincerely invite you to participate in a study: an 8-week treatment with Danlu capsules or placebo in patients with breast hyperplasia accompanied by breast pain, to evaluate the efficacy and safety of Danlu capsules in the clinical treatment of breast hyperplasia with breast pain. This is an exploratory study.

Before you decide whether to participate in this study, please read the following information carefully to fully understand the purpose, procedures, duration, as well as the potential benefits, risks, and discomforts associated with participation. If you have any questions, you may consult your physician at any time. You may also discuss with your family members or friends to help you make a decision.

Study Introduction

I. Background and purpose

Breast hyperplasia is a relatively common condition among women, predominantly affecting patients aged 25 to 50 years, with an incidence rate accounting for 75% of all breast diseases. It causes numerous inconveniences in the lives of patients. The primary clinical features of breast hyperplasia are cyclically worsening breast pain and multiple breast lumps. Although it is often associated with mild self-limiting pain, approximately 15% of affected women still require treatment. Currently, the first-line treatment for breast pain is conservative management, including physical support, over-the-counter analgesics, and adjustment of hormone medications used by the patient. If ineffective, second-line treatments such as tamoxifen may be recommended, but they can also cause menopausal-like symptoms. Therefore, there is an urgent need to explore safer and more effective treatment options.

Danlu capsules (formerly known as Kelutong capsules) is a traditional Chinese medicine preparation for the treatment of breast hyperplasia. Danlu capsules have been approved for marketing by the National Medical Products Administration, obtained a patent, and are recommended by several clinical guidelines and consensus statements as a treatment for breast hyperplasia. Preliminary studies have suggested that Danlu capsules may have some efficacy in relieving symptoms of breast hyperplasia, improving breast pain, and reducing the size of breast lumps. This project will employ a prospective, randomized, double-blind, controlled design, combined with modern laboratory techniques, focusing on improving breast pain symptoms in clinical patients. It is expected to provide new theoretical support for the treatment strategies of fibrocystic breast disease and offer safer and more effective treatment options for patients with breast pain associated with fibrocystic breast disease.

This study will be conducted at the Breast Disease Specialty Hospital of Guangdong Provincial Hospital of Chinese Medicine and multiple collaborating centers, with an estimated enrollment of 264 patients. The study has been approved by the Ethics Committee of Guangdong Provincial

Hospital of Chinese Medicine. The Ethics Committee of Guangdong Provincial Hospital of Chinese Medicine has reviewed and confirmed that this study complies with the principles of the Declaration of Helsinki and is in accordance with medical ethics.

II. Who is suitable to participate in the study
1. You may be eligible to participate in this study if you meet all the following criteria:
(1) Female patients aged 18 to 65 years (inclusive of 18 and 65 years);
(2) Meet the diagnostic criteria for breast hyperplasia according to Western medicine and have a visual analog scale (VAS) score for breast pain ≥ 4;
(3) Voluntarily agree to participate in this study, sign the informed consent form, and have good compliance and willingness to cooperate with follow-up visits.
2. However, you will not be eligible to participate in this study if you have any of the following conditions:
(1) Presence of other breast-related diseases such as acute mastitis, benign or malignant breast tumors;
(2) Presence of diseases causing chest pain due to other causes;
(3) Presence of severe primary diseases of the liver, kidneys, or hematopoietic system;
(4) Biochemical tests indicating total bilirubin (TBIL) > 1.5 × upper limit of normal (ULN), alanine aminotransferase (ALT) > 1.5 × ULN, or aspartate aminotransferase (AST) > 1.5 × ULN;
(5) Pregnant or breastfeeding women;
(6) Individuals with allergic constitution or allergy to the components of the study drug;
(7) Patients with a confirmed diagnosis of anxiety disorder in the past;
(8) Participation in another clinical trial within the past 3 months.

III. What will be required if you participate in the study

1. Before you are enrolled in the study

Prior to your enrollment, you will undergo the following assessments to determine your eligibility for participation. The physician will inquire about and document your medical history and conduct a comprehensive physical examination.

2. If you are eligible, the study will proceed as follows

At the start of the study, you will be randomly assigned to receive either Danlu capsules or placebo based on a random number provided by a computer. Patients participating in this study have a 50% chance of being allocated to either treatment group. Neither you nor your physician will know or be able to choose which treatment you will receive in advance. The treatment group will receive Danlu capsules orally, while the control group will receive placebo orally. The dosing regimen for both groups will be three times daily, once in the morning, once at noon, and once in the evening, with four capsules per dose. The treatment will last for eight weeks, with medication discontinued during menstruation.

Before treatment begins and at week four of treatment: You should visit the hospital and provide the physician with an accurate account of any changes in your condition. The physician will collect your medical history.

Within three days after the end of treatment (week eight): The study will be concluded at this point. You should visit the hospital and provide the physician with an accurate account of any changes in your condition. The physician will collect your medical history and conduct a physical examination.

One month after the end of treatment: To follow up on your condition, you will need to visit the hospital again. The physician will inquire about and document any changes in your condition and conduct a physical examination.

3. Other matters that require your cooperation

You are required to visit the hospital at the times agreed upon with your physician. These follow-up visits are crucial as they enable the physician to assess whether the treatment you received has been effective.

You must take the medication as directed by your physician. During each follow-up visit, you are required to return any unused medication and its packaging. Additionally, you should bring any other medications you are currently taking, including those for any comorbid conditions that you need to continue taking.

During the study period, you must not participate in any other related research. If you require any other treatments, you should consult your physician in advance.

Regarding dietary and lifestyle regulations: You should maintain a regular diet and normal daily routine.

4. Anticipated circumstances and/or reasons for termination of your participation in the trial

Your participation in the study may be terminated or discontinued if any of the following situations occur:

Premature termination: If you experience a severe allergic reaction clearly related to the study medication; if you develop adverse symptoms, signs, or abnormal test results clearly related to the study medication, and the investigator deems it necessary to terminate the study after careful assessment; if you become pregnant during the study period.

Other factors: If you withdraw your informed consent; if you are lost to follow-up; if you use or receive any treatment drugs/other therapies that may affect the observed indicators without authorization.

IV. Potential benefits of participating in the study

You may potentially benefit from participating in this study. These benefits include the possibility of improvement in your condition and the potential contribution to the development of a new treatment method that could be used for other patients with similar conditions.

Your participation will help advance medical research and may bring potential benefits to patients with similar diseases.

V. Potential risks and discomforts of participating in the study

As with any drug treatment, participation in this study may result in some adverse reactions. During this clinical study, if any harm or serious adverse events related to the trial occur, medical treatment will be provided by the participant's physician and the hospital. The main adverse reactions may include: gastrointestinal reactions such as nausea, abdominal pain, vomiting, and epigastric discomfort, with a total incidence rate of 1.66%.

If these symptoms occur, the physician will provide appropriate symptomatic treatment based on your specific condition.

During the study, you will need to visit the hospital for follow-up appointments and undergo some laboratory and physical examinations, which may cause you inconvenience or trouble.

In addition, any treatment may be ineffective, and the condition may continue to progress due to treatment failure or the presence of other comorbidities. These are treatment risks that every patient faces when seeking medical care. Even if you do not participate in this clinical study, these treatment risks would still exist. During the study, if the physician (or investigator) determines that the treatment measures taken in this study are ineffective, the study will be terminated, and other potentially effective treatment measures will be adopted.

VI. Expenses

The research team will cover the consultation fees and examination costs related to the study during your follow-ups, including laboratory tests (complete blood count, urinalysis, liver and kidney function tests, breast ultrasound, etc.) after enrollment, and will provide the study medication free of charge. Participation in this study will not incur any additional costs for you. You will receive a total transportation subsidy of 500 yuan (150 yuan after the second and third follow-ups, and 200 yuan after the final follow-up), calculated based on the actual number of visits and paid in full after the clinical study ends. If any harm related to the trial occurs, the research team will cover your medical expenses and provide appropriate financial compensation in accordance with laws and regulations.

Any treatment and examination costs related to other comorbidities you may have are not included in the free scope.

VII. Confidentiality of personal information

Your medical records (study case report forms/CRFs, laboratory test results, etc.) will be completely stored in the hospital. The physician will record the test results in your outpatient medical record. Investigators, representatives of the sponsor, and the ethics committee will be permitted to access your medical records. Any public reports of the study results will not disclose your personal identity. We will make every effort to protect the privacy of your personal medical information within the limits of the law.

VIII. How to obtain more information

You may ask the investigator any questions about this study at any time. The physician or investigator will leave his/her phone number with you to answer your questions.

If you have any complaints or questions about participating in the study, please contact the Office of the Ethics Committee of Guangdong Provincial Hospital of Chinese Medicine (phone number: 020-81887233-35943).

If there is any significant new information during the study that may affect your willingness to continue participating, your physician will inform you promptly.

IX. Voluntary participation and withdrawal from the study

Your participation in this study is entirely voluntary. You have the right to refuse to participate in this study or to withdraw from it at any time during the study without affecting your relationship with the physician or causing any loss of other medical benefits.

Your physician or investigator may terminate your participation in the study at any time if they believe it is in your best interest.

If you choose not to participate in this study or withdraw from it, there are many alternative treatment options available, such as oral medications. You do not need to participate in this study to treat your condition.

If you withdraw from the study for any reason, you may be asked about your experience with the study treatment. If the physician deems it necessary, you may be required to undergo laboratory tests and a physical examination. This is very beneficial for protecting your health.

X. What to do now

Before you decide to participate in the study, please ask your physician as many questions as possible until you fully understand the study. The decision to participate is yours alone. You may discuss it with your family or friends before making a decision.

Thank you for reading the above information. If you decide to participate in the study, please inform your physician or research assistant, and we will arrange a suitable time for your visit.

Please keep this document for your reference.

Signature Leaflet for Informed Consent

Clinical Research Project Name: Prospective Randomized Double-blind Multicenter Clinical Study of Danlu Capsules for the Treatment of Breast Hyperplasia

Sponsor/Project-issuing Unit: Guangdong Provincial Hospital of Chinese Medicine

Documentation of Task Assignment from the Funding Agency: Available

Ethics Review Approval Number:

Statement of Consent

I have read the above introduction to this study and have had the opportunity to discuss it and ask questions of the investigator. All my questions have been answered to my satisfaction.

I understand the potential risks and benefits of participating in this study. I am aware that my participation is voluntary, and I confirm that I have had ample time to consider this and understand that:

● I can obtain further information from the investigator at any time.

● I may withdraw from this study at any time without any discrimination or reprisal, and my medical treatment and rights will not be affected.

If I need to take any other medication due to illness, I will consult my physician beforehand or inform the investigator truthfully afterward.

I agree to allow the Ethics Committee or representatives of the sponsor to review my study-related information.

I will receive a copy of the signed and dated informed consent form.

Finally, I decide to agree to participate in this study.

Patient/Participant Signature:___________________________ Date: ________________________

Contact Number:

Legal Representative’s Signature (if applicable):_____________________Date: ________________

Contact Information of Legal Representative:

(Note: If the participant is unable to sign the informed consent due to lack of or limited legal capacity, the legal representative will sign on their behalf.)

Witness Signature (if applicable):________________________ Date: ________________________

Contact Information of Witness:

I confirm that I have explained the details of this trial to the patient, including their rights and the potential benefits and risks, and have provided them with a copy of the signed informed consent form.

Investigator’s Signature:______________________________ Date: ______________________

Investigator’s Work Phone Number:

Contact Number for the Office of the Ethics Committee of Guangdong Provincial Hospital of Chinese Medicine: 020–81887233–35943
