# Supplementary material for: Effect of Danlu capsules for the treatment of breast hyperplasia with mastalgia: a multicenter, double-blind, randomized controlled trial protocol
Source: Front Med (Lausanne). 2025 Oct 21;12:1687673. doi: 10.3389/fmed.2025.1687673 (PMC12582957; doi:10.3389/fmed.2025.1687673)
Supplement: Supplementary file 3 [file Data_Sheet_3.pdf]

Project Number：WFCMS2024015

Prospective, Randomized, Double-Blind, Multicenter Clinical Study on Danlu Capsule for the Treatment of Mammary Gland Hyperplasia: Statistical Analysis Plan

Sponsoring Institution: Guangdong Provincial Hospital of Chinese Medicine
Version/Date: 1.0/2025-02-25

Statistical Analysis Center: Center for Drug Clinical Research, Shanghai University of Traditional Chinese Medicine
Statistician: Jihan Huang

Version History of Statistical Analysis Plan

Version Number	Date	Changes	Reason for Revision
1.0	2025-02-25	Initial Version	N/A

Table of Contents

Abbreviations 2
1 Background 3
2 Objectives 3
3 Study Design 3
4 Study Medication and Administration 3
5 Efficacy and Safety Assessments 3
6 Statistical Analysis Methods 4
7 Research Results 7
7.1 Participant Disposition 7
7.2 Demographics and Baseline Characteristics 7
7.3 Medication Compliance and Concomitant Medication Analysis 9
7.4 Efficacy Analysis 9
7.4.1 Breast Pain Analysis 9
7.4.2 Breast Mass Size and Consistency 10
7.4.3 SF-36 Quality of Life Scale Analysis 12
7.4.4 Anxiety and Depression Rating Scale Analysis 17
7.4.5 Sex Hormones 19
7.4.6 Cytokines 20
7.5 Safety Analysis 21
7.5.1 Adverse Event Analysis 21

Abbreviations

Abbreviation	Annotation
ALP	Alkaline Phosphatase
ALT	Alanine Aminotransferase
ANCOVA	Analysis of Covariance
AST	Aspartate Aminotransferase
ATC	Anatomical Therapeutic Chemical Classification System
BMI	Body Mass Index
CDE	Center for Drug Evaluation
CI	Confidence Interval
CMH	Cochran-Mantel-Haenszel Test
Cr	Creatinine
CRF	Case Report Form
CS	Clinically Significant
eCRF	Electronic Case Report Form
FAS	Full Analysis Set
Fisher	Fisher's Exact Probability Test
GAD-7	Generalized Anxiety Disorder-7
HGB	Hemoglobin
LOCF	Last Observation Carried Forward
ITT	Intention-to-treat Analysis
Max	Maximum
Mean	Mean
MedDRA	Medical Dictionary for Regulatory Activities
Median	Median
Min	Minimum
MMRM	Mixed-effect Models for Repeated Measures
NCS	Not Clinically Significant
NMPA	National Medical Products Adiministration
PHQ-9	Patient Health Questionnaire-9
PLT	Platelets
PPS	Per Protocol Set
PT	Preferred Term
RBC	Red Blood Cells
SADR	Serious Adverse Drug Reaction
SAE	Serious Adverse Event
SAS	Self-Rating Anxiety Scale
Scr	Serum Creatinine
SD	Standard Deviation
SDS	Self-Rating Depression Scale
SF-36	Quality of Life Scale
SOC	System Organ Class
SS	Safety Set
TBIL	Total Bilirubin
TEAE	Treatment-Emergent Adverse Event
VAS	Visual analog scale
WBC	White Blood Cells
WHO	World Health Organization
γ-GT	Gamma-Glutamyl Transferase

Prospective, Randomized, Double-Blind, Multicenter Clinical Study on Danlu Capsule for the Treatment of Mammary Gland Hyperplasia: Statistical Analysis Plan

1 Background

Breast hyperplasia with breast pain is relatively common among women. Although it is usually associated with mild self-limiting pain, approximately 15% of affected women still require treatment. The current first-line treatment for breast pain is conservative therapy, including physical support, over-the-counter analgesics, and adjustment of hormonal medications used by the patient. When this is ineffective, second-line treatments such as tamoxifen may be recommended, but they can also cause menopausal-like symptoms. Therefore, there is an urgent need to explore safer and more effective treatment options. Danlu Capsule is a traditional Chinese medicine preparation for the treatment of breast hyperplasia. Previous studies have suggested that it has certain therapeutic effects in relieving symptoms of breast hyperplasia, improving breast pain, and reducing the size of breast lumps. However, no prospective randomized controlled double-blind study has been conducted to further clarify the clinical efficacy, safety, and mechanism of action of Danlu Capsule in treating breast pain.

This project focuses on the prominent issue of the lack of a standard and safe treatment for breast hyperplasia with breast pain. It aims to improve the breast pain symptoms of clinical patients by conducting a multicenter prospective randomized double-blind controlled study to clarify the clinical efficacy of Danlu capsule in treating breast hyperplasia with breast pain. This will provide an effective and safe clinical treatment option for patients with breast hyperplasia and breast pain.

This statistical analysis plan is developed based on the protocol of the "Prospective Randomized Double-Blind Multicenter Clinical Study of Danlu Capsule for the Treatment of Breast Hyperplasia" (Project Number: WFCMS2024015) and the case report form (CRF version date: February 20, 2025, CRF version number: 1.0). The statistical analysis plan will be finalized before the database lock of this study.

2 Objectives

To evaluate the efficacy and safety of Danlu capsule in the treatment of breast hyperplasia.

3 Study Design

A randomized, double-blind, placebo-controlled, multicenter clinical study design will be adopted, and it is expected to be conducted concurrently at ten research centers across the country.

4 Study Medication and Administration

4.1 Study Medications

- Treatment medication: Danlu capsule; manufacturer: Jiangsu Sino-Pharmaceutical Group Co., Ltd.; specification: 0.5 g per capsule; dosage: 2.0 g per dose, three times per day.
- Placebo medication: Danlu capsule placebo; manufacturer: Jiangsu Sino-Pharmaceutical Group Co., Ltd.; specification: 0.5 g per capsule; dosage: 2.0 g per dose, three times per day.

4.2 Treatment Duration

The treatment period is eight weeks, followed by a one-month follow-up after discontinuation of the medication.

4.3 Concomitant Medications

During the entire treatment period, the use of other Western medicines, traditional Chinese medicines, patent Chinese medicines, or external therapies with similar functions and indications as the study medication is prohibited. If any other non-prohibited Western medicines, traditional Chinese medicines, or patent Chinese medicines have been used, their names, reasons for use, dosage, and duration of treatment should be recorded in detail on the case observation form.

5 Efficacy and Safety Assessments

5.1 Efficacy Evaluation

5.1.1 Primary Efficacy Endpoint
Breast pain assessment: The visual analog scale (VAS) will be used to assess the severity of breast pain before and after treatment. The scale ranges from 0 to 10, with 0 indicating no pain and 10 indicating severe pain. Higher scores indicate more severe pain. The primary outcome is defined as the change in the breast pain VAS score from baseline to the 8-week time point.
5.1.2 Secondary Efficacy Endpoints
(1) Quality of life scale (SF-36)
The SF-36 scale consists of 36 items and evaluates eight dimensions of health-related quality of life: physical functioning, role-physical, bodily pain, general health, vitality, social functioning, role-emotional, and mental health. Assessments will be conducted at baseline and after treatment.
(2) Anxiety and depression assessment scales
The patient health questionnaire-9 (PHQ-9), generalized anxiety disorder-7 (GAD-7), self-rating depression scale (SDS), and self-rating anxiety scale (SAS) will be administered at baseline and after pharmacological intervention to assess the levels of anxiety and depression in patients.
(3) Breast lump changes
Professional ultrasound physicians will use breast ultrasound and other examinations to measure the size, number, and texture of breast lumps and assess the changes in these lumps before and after treatment.
(4) Hormonal regulation indicators
Serum levels of estrogen, progesterone, prolactin, and other hormones will be measured before and after treatment to evaluate hormonal changes.
(5) Cytokines
Levels of interleukin-6 (IL-6), tumor necrosis factor-alpha (TNF-α), and other cytokines will be measured before and after treatment to assess changes.
5.2 Safety Evaluation
(1) Incidence of adverse events/adverse reactions.
(2) Laboratory tests (complete blood count, hepatic and renal function).
6 Statistical Analysis Methods
6.1 Hypothesis Testing
Null hypothesis H_0: $P_1 - P_2 = 0$,
Alternative hypothesis H_1: $P_1 - P_2 \neq 0$,
Where P_1 is the mean of the treatment group and P_2 is the mean of the control group.
6.2 Sample Size Estimation
Based on the literature (Jiangsu Journal of Traditional Chinese Medicine, 2021, 53(8): 3 and Journal of Traditional Chinese Medicine, 2019(17): 5), the reduction in breast pain VAS score after two menstrual cycles of treatment with Danlu capsule is 3.2 points, while the reduction in the control group (placebo) is approximately 1.5 points, with a standard deviation of 3.4 points. The sample size for this superiority trial was calculated using a t-test. Based on a superiority margin of 0, a two-sided α of 0.05, and β of 0.05 (power = 95%), with 1:1 allocation, the required sample size per group was determined to be 105 using PASS 21.0 software. Considering a dropout rate of 20%, the sample size is increased to 132 cases per group, resulting in a total of 264 cases.
6.3 Statistical Analysis Data Sets
(1) Full analysis set (FAS): The collection of all cases that were randomized and received at least one dose of the study medication. The primary study conclusions are based on the results of the FAS analysis.
(2) Per-protocol set (PPS): A dataset generated by participants who fully complied with the study protocol. Compliance includes the treatment received, the availability of measurements for the primary endpoint, and no major deviations from the study protocol. The PPS analysis is used for the primary efficacy endpoint.

(3) Safety set (SS): The actual data of participants who received at least one treatment and have post-treatment safety indicator records. The incidence of adverse reactions is calculated using the number of cases in the SS as the denominator.

6.4 Content and Methods of Statistical Analysis

6.4.1 General Principles of Statistical Analysis

(1) Statistical analysis software: SAS 9.4 software will be used for statistical analysis.

(2) Statistical tests: All statistical tests will be two-sided. A p-value less than or equal to 0.05 will be considered to indicate a statistically significant difference.

6.4.2 Handling of Missing Values

For missing values of primary outcomes in efficacy analyses: the last observation carried forward method will be adopted for imputation in the primary analysis, and multiple imputation will be used for the missing primary outcome data as a sensitivity analysis to verify result robustness.

6.4.3 Distribution of Participants

- The number of participants screened, randomized, and completed the trial will be listed for the overall study and each center to determine the three analysis datasets (FAS, PPS, SS).
- The number and proportion of participants screened, participants who failed screening, and reasons for failure will be listed for the overall study and each center.

6.4.4 Demographic Data and Baseline Analysis

Analysis will be based on the actual data in the FAS.

Descriptive statistics for demographic data and other baseline characteristics:

- For continuous variables that follow a normal distribution, the number of cases, mean, and standard deviation will be calculated; for those that do not follow a normal distribution, the number of cases, median, and quartiles will be calculated.
- For categorical and ordinal data, the frequency and proportion will be calculated.
- Inferential statistical results (p-values) will be listed as descriptive results.

6.4.5 Medication Adherence and Concomitant Medication Analysis

6.4.5.1 Medication Adherence Analysis

- The percentage of participants with medication adherence within the range of 80%-120% will be calculated, and differences between groups will be compared using the chi-square test or Fisher’s exact test.
- Medication exposure will be compared between groups using the t-test.
- A table will be used to describe the actual medication intake, prescribed medication intake, and medication adherence of participants.

6.4.5.2 Concomitant Medication Analysis

- Concomitant medications will be coded using World Health Organization (WHO) Drug and summarized according to the first level of Anatomical Therapeutic Chemical Classification System (ATC) classification, with the number of cases and proportions calculated.
- A list of concomitant medications will be provided.

6.4.6 Efficacy Analysis

Primary Efficacy Endpoint Analysis:

The difference between the breast pain VAS score at eight week and the baseline value will be analyzed using Analysis of Covariance (ANCOVA), with treatment group and center as fixed effects, and baseline value as a covariate. Missing values will be imputed using the LOCF method. Both PPS and FAS analyses will be conducted. Changes in breast pain VAS score from baseline at weeks 4, 8, and 12 (1 month after discontinuation of treatment) will be compared between groups using mixed-effects model for repeated measures (MMRM), with the model including treatment group, visit, group-by-visit interaction, and baseline value as a covariate. Missing values will not be imputed.

Secondary Efficacy Endpoint Analysis:

(1) Quality of life scale (SF-36) Score Changes at weeks 4, 8, and 12 (one month after discontinuation of treatment):

Group differences will be compared using MMRM, with the model including treatment group, visit, group-by-visit interaction, and baseline value as a covariate. Missing values will not be imputed. The analysis will be based on the actual data in the FAS.

(2) Anxiety and depression assessment scale score changes at weeks 4, 8, and 12 (one month after discontinuation of treatment):

Group differences will be compared using MMRM, with the model including treatment group, visit, group-by-visit interaction, and baseline value as a covariate. Missing values will not be imputed. The analysis will be based on the actual data in the FAS.

(3) Breast lump size changes at weeks 4 and 12 (one month after discontinuation of treatment):

Group differences will be compared using MMRM, with the model including treatment group, visit, group-by-visit interaction, and baseline value as a covariate. Missing values will not be imputed. The analysis will be based on the actual data in the FAS.

(4) Hormonal regulation indicators (serum estrogen, progesterone, prolactin) changes:

Group differences will be compared using the Wilcoxon test. The analysis will be based on the actual data in the FAS.

(5) Cytokine (IL-6, TNF-α) changes:

Group differences will be compared using the Wilcoxon test. The analysis will be based on the actual data in the FAS.

6.4.7 Safety Analysis

6.4.7.1 Adverse Events

- Causality assessment between adverse events and the investigational product will be performed in strict accordance with the five-category classification (definitely related, probably related, possibly related, probably unrelated, and unrelated) as specified in the Technical Guidelines for Evaluation of the Correlation of Adverse Events in Drug Clinical Trials (Trial Implementation) issued by the Center for Drug Evaluation (CDE) of the National Medical Products Administration (NMPA) in 2024.
- Adverse events occurring before the first dose of the study drug after signing the informed consent form will be defined as pre-treatment adverse events (AEs).
- Treatment-emergent adverse events (TEAEs) are defined as adverse events that occur or worsen after the first dose of the study drug.
- Adverse reactions are defined as adverse events with a relationship to the study drug classified as “definitely related”, “probably related”, or “possibly related”.
- TEAEs will be summarized by the following categories:
  - Adverse events/reactions
  - Serious adverse events/reactions
  - Adverse events/reactions leading to withdrawal from the study
- Adverse events will be summarized by System Organ Class (SOC) and Preferred Term (PT), with the number of cases, number of occurrences, and incidence rates calculated.
- A detailed list of all adverse events will be provided.

6.4.7.2 Laboratory Tests

- Cross-tabulations of laboratory test results before and after treatment for complete blood count (RBC, HGB, WBC, PLT), hepatic function (ALT, AST, γ-GT, ALP, TBIL), and renal function (BUN/UREA and Cr) will be listed.
- Descriptive statistics for changes from baseline and actual values of laboratory tests at each visit for complete blood count (RBC, HGB, WBC, PLT), hepatic function (ALT, AST, γ-GT, ALP, TBIL), and renal function (BUN/UREA and Cr) will be provided.
- A list of participants with normal baseline laboratory test values that became abnormal after treatment, including test values and clinical significance assessments, will be provided.

- A list of participants with abnormal baseline laboratory test values that were not clinically significant but became clinically significant after treatment, including test values and clinical significance assessments, will be provided.

6.5 Interim Analysis

No interim analysis will be conducted in this study.

7 Research Results

7.1 Participant Disposition

Table 1-1 Distribution of participants by center (randomized participants)

	Treatment group (N=XXX)	Placebo group (N=XXX)	Total (N=XXX)
Randomized	XXX	XXX	XXX
Completed	XXX	XXX	XXX
FAS	XXX	XXX	XXX
PPS	XXX	XXX	XXX
SS	XXX	XXX	XXX

Table 1-2 Classification of participant completion (randomized participants)

Indicator	Treatment group (N=XXX)	Placebo group (N=XXX)	Total (N=XXX)
Randomized (N)	XXX	XXX	XXX
Completed the trial n (%)	XXX (XX.X)	XXX (XX.X)	XXX (XX.X)
Participants discontinued the trial n (%)	XXX (XX.X)	XXX (XX.X)	XXX (XX.X)
Reasons for discontinuation			
Lost to follow-up n (%)	XX (XX.X)	XX (XX.X)	XX (XX.X)
Participant requested withdrawal n (%)	XX (XX.X)	XX (XX.X)	XX (XX.X)
Due to serious adverse event, please specify SAE n (%)	XX (XX.X)	XX (XX.X)	XX (XX.X)
Development of serious concomitant disease n (%)	XX (XX.X)	XX (XX.X)	XX (XX.X)
Significant protocol deviation in medication use n (%)	XX (XX.X)	XX (XX.X)	XX (XX.X)
Pregnancy during treatment n (%)	XX (XX.X)	XX (XX.X)	XX (XX.X)
Other reasons n (%)	XX (XX.X)	XX (XX.X)	XX (XX.X)

7.2 Demographics and Baseline Characteristics

Table 2-1 Demographic data analysis (FAS)

Indicator	Treatment group (N=XXX)	Placebo group (N=XXX)	Total (N=XXX)
Age (years), Mean±SD	XX.XX±XX.XX	XX.XX±XX.XX	XX.XX±XX.XX
Gender			
Male n (%)	XXX (XX.X)	XXX (XX.X)	XXX (XX.X)
Female n (%)	XXX (XX.X)	XXX (XX.X)	XXX (XX.X)
Ethnicity			
Han n (%)	XXX (XX.X)	XXX (XX.X)	XXX (XX.X)
Non-Han n (%)	XXX (XX.X)	XXX (XX.X)	XXX (XX.X)
Height (cm), Mean±SD	XX.XX±XX.XX	XX.XX±XX.XX	XX.XX±XX.XX
Weight (kg), Mean±SD	XX.XX±XX.XX	XX.XX±XX.XX	XX.XX±XX.XX
BMI (kg/m^2), Mean±SD	XX.XX±XX.XX	XX.XX±XX.XX	XX.XX±XX.XX
Duration of disease (months), median (IQR)	XX.X (XX.X, XX.X)	XX.X (XX.X, XX.X)	XX.X (XX.X, XX.X)

Indicator	Treatment group (N=XXX)	Placebo group (N=XXX)	Total (N=XXX)
Comorbidities or symptoms n (%)	XXX (XX.X)	XXX (XX.X)	XXX (XX.X)

Table 2-2 Menstrual history and past medical history (FAS)

Indicator	Treatment group (N=XXX)	Placebo group (N=XXX)	Total (N=XXX)
Age at menarche (years), median (IQR)	XX.X (XX.X, XX.X)	XX.X (XX.X, XX.X)	XX.X (XX.X, XX.X)
Menstrual cycle (days), median (IQR)	XX.X (XX.X, XX.X)	XX.X (XX.X, XX.X)	XX.X (XX.X, XX.X)
Menstrual period (days), median (IQR)	XX.X (XX.X, XX.X)	XX.X (XX.X, XX.X)	XX.X (XX.X, XX.X)
Number of pregnancies (times), median (IQR)	XX.X (XX.X, XX.X)	XX.X (XX.X, XX.X)	XX.X (XX.X, XX.X)
Number of abortions (times), median (IQR)	XX.X (XX.X, XX.X)	XX.X (XX.X, XX.X)	XX.X (XX.X, XX.X)
Breastfeeding history			
No n (%)	XXX (XX.X)	XXX (XX.X)	XXX (XX.X)
Yes n (%)	XXX (XX.X)	XXX (XX.X)	XXX (XX.X)
Duration of breastfeeding (months), median (IQR)	XX.X (XX.X, XX.X)	XX.X (XX.X, XX.X)	XX.X (XX.X, XX.X)
Mastitis			
No n (%)	XXX (XX.X)	XXX (XX.X)	XXX (XX.X)
Yes n (%)	XXX (XX.X)	XXX (XX.X)	XXX (XX.X)
History of breast surgery			
No n (%)	XXX (XX.X)	XXX (XX.X)	XXX (XX.X)
Yes n (%)	XXX (XX.X)	XXX (XX.X)	XXX (XX.X)
Medication history			
No n (%)	XXX (XX.X)	XXX (XX.X)	XXX (XX.X)
Yes n (%)	XXX (XX.X)	XXX (XX.X)	XXX (XX.X)

Table 2-3 Breast pain, breast lump scores, quality of life scale, and anxiety and depression assessment scales (FAS)

Indicator	Treatment group (N=XXX)	Placebo group (N=XXX)	Total (N=XXX)
Breast pain, Mean±SD	XX.XX±XX.XX	XX.XX±XX.XX	XX.XX±XX.XX
Longest diameter size (cm), Mean±SD	XX.XX±XX.XX	XX.XX±XX.XX	XX.XX±XX.XX
Longest diameter size score, Mean±SD	XX.XX±XX.XX	XX.XX±XX.XX	XX.XX±XX.XX
Consistency score, Mean±SD	XX.XX±XX.XX	XX.XX±XX.XX	XX.XX±XX.XX
SF-36			
Physical functioning score, Mean±SD	XX.XX±XX.XX	XX.XX±XX.XX	XX.XX±XX.XX
Role-physical, Mean±SD	XX.XX±XX.XX	XX.XX±XX.XX	XX.XX±XX.XX
Bodily pain, Mean±SD	XX.XX±XX.XX	XX.XX±XX.XX	XX.XX±XX.XX
General health, Mean±SD	XX.XX±XX.XX	XX.XX±XX.XX	XX.XX±XX.XX
Vitality, Mean±SD	XX.XX±XX.XX	XX.XX±XX.XX	XX.XX±XX.XX
Social functioning, Mean±SD	XX.XX±XX.XX	XX.XX±XX.XX	XX.XX±XX.XX
Role-emotional, Mean±SD	XX.XX±XX.XX	XX.XX±XX.XX	XX.XX±XX.XX
Mental health, Mean±SD	XX.XX±XX.XX	XX.XX±XX.XX	XX.XX±XX.XX
Anxiety and depression assessment scales			
PHQ-9 score, Mean±SD	XX.XX±XX.XX	XX.XX±XX.XX	XX.XX±XX.XX
GAD-7 score, Mean±SD	XX.XX±XX.XX	XX.XX±XX.XX	XX.XX±XX.XX
Self-rating depression scale (SDS) score, Mean±SD	XX.XX±XX.XX	XX.XX±XX.XX	XX.XX±XX.XX
Self-rating anxiety scale (SAS) score, Mean±SD	XX.XX±XX.XX	XX.XX±XX.XX	XX.XX±XX.XX

Table 2-4 Hormonal regulation and cytokine indicators (FAS)

Indicator	Treatment group (N=XXX)	Placebo group (N=XXX)	Total (N=XXX)
Estrogen (pg/ml), median (IQR)	XX.X (XX.X, XX.X)	XX.X (XX.X, XX.X)	XX.X (XX.X, XX.X)
Progesterone (nmol/L), median (IQR)	XX.X (XX.X, XX.X)	XX.X (XX.X, XX.X)	XX.X (XX.X, XX.X)
Prolactin (ng/ml), median (IQR)	XX.X (XX.X, XX.X)	XX.X (XX.X, XX.X)	XX.X (XX.X, XX.X)
IL-6 (pm/ml), median (IQR)	XX.X (XX.X, XX.X)	XX.X (XX.X, XX.X)	XX.X (XX.X, XX.X)
TNF-α (pm/ml), median (IQR)	XX.X (XX.X, XX.X)	XX.X (XX.X, XX.X)	XX.X (XX.X, XX.X)

7.3 Medication Compliance and Concomitant Medication Analysis

Table 3-1 Medication adherence and exposure analysis (FAS)

Indicator	Treatment group (N=XXX)	Placebo group (N=XXX)	Total (N=XXX)
Medication adherence			
Within 80%-120% range n (%)	XXX (XX.X)	XXX (XX.X)	XXX (XX.X)
Outside 80%-120% range n (%)	XXX (XX.X)	XXX (XX.X)	XXX (XX.X)
Medication Exposure (capsules), Median (IQR)	XX.X (XX.X, XX.X)	XX.X (XX.X, XX.X)	XX.X (XX.X, XX.X)

Table 3-2 Concomitant medications coded by WHO Drug Classification Analysis (FAS)

ATC First-Level Classification	Treatment group (N=XXX)		Placebo group (N=XXX)	
ATC Second-Level Classification	Number of cases	Number of occurrences	Number of cases	Number of occurrences
Preferred Term				
N (Nmiss)	XXX		XXX	
Summary n (%)	XX (XX.X)	XXX	XX (XX.X)	XXX
ATC1 n (%)	XX (XX.X)	XXX	XX (XX.X)	XXX
ATC2 n (%)	XX (XX.X)	XXX	XX (XX.X)	XXX
PT n (%)	XX (XX.X)	XXX	XX (XX.X)	XXX
…	XX (XX.X)	XXX	XX (XX.X)	XXX
…	XX (XX.X)	XXX	XX (XX.X)	XXX

7.4 Efficacy Analysis

7.4.1 Breast Pain Analysis

Table 4-1 Analysis of covariance (ANCOVA) of change in breast pain score at week 8 (full analysis set, FAS)

Table 4-2 Analysis of covariance (ANCOVA) of change in breast pain score at week 8 (per protocol set, PPS)

Visit	Treatment group (N=XXX)	Placebo group (N=XXX)
Baseline		
N (NMissing)	XXX	XXX
Mean±SD	XX.XX±XX.XX	XX.XX±XX.XX
8 week		
N (NMissing)	XXX	XXX
Mean±SD	XX.XX±XX.XX	XX.XX±XX.XX
Change from baseline		
N (NMissing)	XXX	XXX
Mean±SD	XX.XX±XX.XX	XX.XX±XX.XX
LS mean (SE)	XX.XX (XX.XX)	XX.XX (XX.XX)
LS mean difference (95% CI)	XX.XX (XX.XX, XX.XX)	
P-value	XX.XX	

Note: Analysis of covariance (ANCOVA) was used, with treatment group and center as fixed effects, and baseline value as a covariate. Missing values were imputed using the last observation carried forward (LOCF) method.

Table 4-3 Temporal comparison of between-group differences in LSMEANS of change in breast pain score by

MMRM (full analysis set, FAS)

Table 4-4 Temporal comparison of between-group differences in LSMEANS of change in breast pain score by MMRM (per protocol set, PPS)

	Treatment group (N=XXX)	Placebo group (N=XXX)
Baseline		
N (NMissing)	XXX	XXX
Mean±SD	XX.XX±XX.XX	XX.XX±XX.XX
4 week		
N (NMissing)	XXX	XXX
Mean±SD	XX.XX±XX.XX	XX.XX±XX.XX
Change from baseline		
N (NMissing)	XXX	XXX
Mean±SD	XX.XX±XX.XX	XX.XX±XX.XX
LS mean (SE)	XX.XX (XX.XX)	XX.XX (XX.XX)
LS mean difference (95% CI)	XX.XX (XX.XX~XX.XX)	
P-value	XX.XX	
8 week		
N (NMissing)	XXX	XXX
Mean±SD	XX.XX±XX.XX	XX.XX±XX.XX
Change from baseline		
N (NMissing)	XXX	XXX
Mean±SD	XX.XX±XX.XX	XX.XX±XX.XX
LS mean (SE)	XX.XX (XX.XX)	XX.XX (XX.XX)
LS mean difference (95% CI)	XX.XX (XX.XX~XX.XX)	
P-value	XX.XX	
12 week		
N (NMissing)	XXX	XXX
Mean±SD	XX.XX±XX.XX	XX.XX±XX.XX
Change from baseline		
N (NMissing)	XXX	XXX
Mean±SD	XX.XX±XX.XX	XX.XX±XX.XX
LS mean (SE)	XX.XX (XX.XX)	XX.XX (XX.XX)
LS mean difference (95% CI)	XX.XX (XX.XX~XX.XX)	
P-value	XX.XX	

Note: Mixed-effects model for repeated measures (MMRM) was used to compare between-group differences, with the model including treatment group, visit, group-by-visit interaction, and baseline value as a covariate. Missing values were not imputed.

7.4.2 Breast Mass Size and Consistency

Table 4-5 Temporal comparison of between-group differences in LSMEANS of change in breast lump longest diameter size (cm) by MMRM (full analysis set, FAS)

	Treatment group (N=XXX)	Placebo group (N=XXX)
Baseline		
N (NMissing)	XXX	XXX
Mean±SD	XX.XX±XX.XX	XX.XX±XX.XX
8 week		
N (NMissing)	XXX	XXX
Mean±SD	XX.XX±XX.XX	XX.XX±XX.XX
Change from baseline		
N (NMissing)	XXX	XXX
Mean±SD	XX.XX±XX.XX	XX.XX±XX.XX
LS mean (SE)	XX.XX (XX.XX)	XX.XX (XX.XX)
LS mean difference (95% CI)	XX.XX (XX.XX~XX.XX)	
P-value	XX.XX	
12 week		

	Treatment group (N=XXX)	Placebo group (N=XXX)
N (NMissing)	XXX	XXX
Mean±SD	XX.XX±XX.XX	XX.XX±XX.XX
Change from baseline		
N (NMissing)	XXX	XXX
Mean±SD	XX.XX±XX.XX	XX.XX±XX.XX
LS mean (SE)	XX.XX (XX.XX)	XX.XX (XX.XX)
LS mean difference (95% CI)	XX.XX (XX.XX~XX.XX)	
P-value	XX.XX	

Note: Mixed-effects model for repeated measures (MMRM) was used to compare between-group differences. The model included treatment group, visit, group-by-visit interaction, and baseline value as a covariate. Missing values were not imputed.

Table 4-6 Temporal comparison of between-group differences in LSMEANS of change in breast lump longest diameter size score by MMRM (full analysis set, FAS)

	Treatment group (N=XXX)	Placebo group (N=XXX)
Baseline		
N (NMissing)	XXX	XXX
Mean±SD	XX.XX±XX.XX	XX.XX±XX.XX
8 week		
N (NMissing)	XXX	XXX
Mean±SD	XX.XX±XX.XX	XX.XX±XX.XX
Change from baseline		
N (NMissing)	XXX	XXX
Mean±SD	XX.XX±XX.XX	XX.XX±XX.XX
LS mean (SE)	XX.XX (XX.XX)	XX.XX (XX.XX)
LS mean difference (95% CI)	XX.XX (XX.XX~XX.XX)	
P-value	XX.XX	
12 week		
N (NMissing)	XXX	XXX
Mean±SD	XX.XX±XX.XX	XX.XX±XX.XX
Change from baseline		
N (NMissing)	XXX	XXX
Mean±SD	XX.XX±XX.XX	XX.XX±XX.XX
LS mean (SE)	XX.XX (XX.XX)	XX.XX (XX.XX)
LS mean difference (95% CI)	XX.XX (XX.XX~XX.XX)	
P-value	XX.XX	

Note: Mixed-effects model for repeated measures (MMRM) was used to compare between-group differences. The model included treatment group, visit, group-by-visit interaction, and baseline value as a covariate. Missing values were not imputed.

Table 4-7 Temporal comparison of between-group differences in LSMEANS of change in breast lump consistency score by MMRM (full analysis set, FAS)

	Treatment group (N=XXX)	Placebo group (N=XXX)
Baseline		
N (NMissing)	XXX	XXX
Mean±SD	XX.XX±XX.XX	XX.XX±XX.XX
8 week		
N (NMissing)	XXX	XXX
Mean±SD	XX.XX±XX.XX	XX.XX±XX.XX
Change from baseline		
N (NMissing)	XXX	XXX
Mean±SD	XX.XX±XX.XX	XX.XX±XX.XX
LS mean (SE)	XX.XX (XX.XX)	XX.XX (XX.XX)
LS mean difference (95% CI)	XX.XX (XX.XX~XX.XX)	

	Treatment group (N=XXX)	Placebo group (N=XXX)
P-value	XX.XX	
12 week		
N (NMissing)	XXX	XXX
Mean±SD	XX.XX±XX.XX	XX.XX±XX.XX
Change from baseline		
N (NMissing)	XXX	XXX
Mean±SD	XX.XX±XX.XX	XX.XX±XX.XX
LS mean (SE)	XX.XX (XX.XX)	XX.XX (XX.XX)
LS mean difference (95% CI)	XX.XX (XX.XX~XX.XX)	
P-value	XX.XX	

Note: Between-group differences were compared using mixed-effects model for repeated measures (MMRM). The model included treatment group, visit, group-by-visit interaction, and baseline value as a covariate. Missing values were not imputed.

7.4.3 SF-36 Quality of Life Scale Analysis

Table 4-8 Temporal comparison of between-group differences in LSMEANS of change in physical functioning score by MMRM (full analysis set, FAS)

	Treatment group (N=XXX)	Placebo group (N=XXX)
Baseline		
N (NMissing)	XXX	XXX
Mean±SD	XX.XX±XX.XX	XX.XX±XX.XX
4 week		
N (NMissing)	XXX	XXX
Mean±SD	XX.XX±XX.XX	XX.XX±XX.XX
Change from baseline		
N (NMissing)	XXX	XXX
Mean±SD	XX.XX±XX.XX	XX.XX±XX.XX
LS mean (SE)	XX.XX (XX.XX)	XX.XX (XX.XX)
LS mean difference (95% CI)	XX.XX (XX.XX~XX.XX)	
P-value	XX.XX	
8 week		
N (NMissing)	XXX	XXX
Mean±SD	XX.XX±XX.XX	XX.XX±XX.XX
Change from baseline		
N (NMissing)	XXX	XXX
Mean±SD	XX.XX±XX.XX	XX.XX±XX.XX
LS mean (SE)	XX.XX (XX.XX)	XX.XX (XX.XX)
LS mean difference (95% CI)	XX.XX (XX.XX~XX.XX)	
P-value	XX.XX	
12 week		
N (NMissing)	XXX	XXX
Mean±SD	XX.XX±XX.XX	XX.XX±XX.XX
Change from baseline		
N (NMissing)	XXX	XXX
Mean±SD	XX.XX±XX.XX	XX.XX±XX.XX
LS mean (SE)	XX.XX (XX.XX)	XX.XX (XX.XX)
LS mean difference (95% CI)	XX.XX (XX.XX~XX.XX)	
P-value	XX.XX	

Note: Between-group differences were compared using Mixed-Effects Model for Repeated Measures (MMRM). The model included treatment group, visit, group-by-visit interaction, and baseline value as a covariate. Missing values were not imputed.

Table 4-9 Temporal comparison of between-group differences in LSMEANS of change in role-physical score by MMRM (full analysis set, FAS)

	Treatment group (N=XXX)	Placebo group (N=XXX)
Baseline		
N (NMissing)	XXX	XXX
Mean±SD	XX.XX±XX.XX	XX.XX±XX.XX
4 week		
N (NMissing)	XXX	XXX
Mean±SD	XX.XX±XX.XX	XX.XX±XX.XX
Change from baseline		
N (NMissing)	XXX	XXX
Mean±SD	XX.XX±XX.XX	XX.XX±XX.XX
LS mean (SE)	XX.XX (XX.XX)	XX.XX (XX.XX)
LS mean difference (95% CI)	XX.XX (XX.XX~XX.XX)	
P-value	XX.XX	
8 week		
N (NMissing)	XXX	XXX
Mean±SD	XX.XX±XX.XX	XX.XX±XX.XX
Change from baseline		
N (NMissing)	XXX	XXX
Mean±SD	XX.XX±XX.XX	XX.XX±XX.XX
LS mean (SE)	XX.XX (XX.XX)	XX.XX (XX.XX)
LS mean difference (95% CI)	XX.XX (XX.XX~XX.XX)	
P-value	XX.XX	
12 week		
N (NMissing)	XXX	XXX
Mean±SD	XX.XX±XX.XX	XX.XX±XX.XX
Change from baseline		
N (NMissing)	XXX	XXX
Mean±SD	XX.XX±XX.XX	XX.XX±XX.XX
LS mean (SE)	XX.XX (XX.XX)	XX.XX (XX.XX)
LS mean difference (95% CI)	XX.XX (XX.XX~XX.XX)	
P-value	XX.XX	

Note: Between-group differences were compared using mixed-effects model for repeated measures (MMRM). The model included treatment group, visit, group-by-visit interaction, and baseline value as a covariate. Missing values were not imputed.

Table 4-10 Temporal comparison of between-group differences in LSMEANS of change in bodily pain score by MMRM (full analysis set, FAS)

	Treatment group (N=XXX)	Placebo group (N=XXX)
Baseline		
N (NMissing)	XXX	XXX
Mean±SD	XX.XX±XX.XX	XX.XX±XX.XX
4 week		
N (NMissing)	XXX	XXX
Mean±SD	XX.XX±XX.XX	XX.XX±XX.XX
Change from baseline		
N (NMissing)	XXX	XXX
Mean±SD	XX.XX±XX.XX	XX.XX±XX.XX
LS mean (SE)	XX.XX (XX.XX)	XX.XX (XX.XX)
LS mean difference (95% CI)	XX.XX (XX.XX~XX.XX)	
P-value	XX.XX	
8 week		
N (NMissing)	XXX	XXX
Mean±SD	XX.XX±XX.XX	XX.XX±XX.XX
Change from baseline		
N (NMissing)	XXX	XXX

	Treatment group (N=XXX)	Placebo group (N=XXX)
Mean±SD	XX.XX±XX.XX	XX.XX±XX.XX
LS mean (SE)	XX.XX (XX.XX)	XX.XX (XX.XX)
LS mean difference (95% CI)	XX.XX (XX.XX~XX.XX)	
P-value	XX.XX	
12 week		
N (NMissing)	XXX	XXX
Mean±SD	XX.XX±XX.XX	XX.XX±XX.XX
Change from baseline		
N (NMissing)	XXX	XXX
Mean±SD	XX.XX±XX.XX	XX.XX±XX.XX
LS mean (SE)	XX.XX (XX.XX)	XX.XX (XX.XX)
LS mean difference (95% CI)	XX.XX (XX.XX~XX.XX)	
P-value	XX.XX	

Note: Between-group differences were compared using mixed-effects model for repeated measures (MMRM). The model included treatment group, visit, group-by-visit interaction, and baseline value as a covariate. Missing values were not imputed.

Table 4-11 Temporal comparison of between-group differences in LSMEANS of change in general health score by MMRM (full analysis set, FAS)

	Treatment group (N=XXX)	Placebo group (N=XXX)
Baseline		
N (NMissing)	XXX	XXX
Mean±SD	XX.XX±XX.XX	XX.XX±XX.XX
4 week		
N (NMissing)	XXX	XXX
Mean±SD	XX.XX±XX.XX	XX.XX±XX.XX
Change from baseline		
N (NMissing)	XXX	XXX
Mean±SD	XX.XX±XX.XX	XX.XX±XX.XX
LS mean (SE)	XX.XX (XX.XX)	XX.XX (XX.XX)
LS mean difference (95% CI)	XX.XX (XX.XX~XX.XX)	
P-value	XX.XX	
8 week		
N (NMissing)	XXX	XXX
Mean±SD	XX.XX±XX.XX	XX.XX±XX.XX
Change from baseline		
N (NMissing)	XXX	XXX
Mean±SD	XX.XX±XX.XX	XX.XX±XX.XX
LS mean (SE)	XX.XX (XX.XX)	XX.XX (XX.XX)
LS mean difference (95% CI)	XX.XX (XX.XX~XX.XX)	
P-value	XX.XX	
12 week		
N (NMissing)	XXX	XXX
Mean±SD	XX.XX±XX.XX	XX.XX±XX.XX
Change from baseline		
N (NMissing)	XXX	XXX
Mean±SD	XX.XX±XX.XX	XX.XX±XX.XX
LS mean (SE)	XX.XX (XX.XX)	XX.XX (XX.XX)
LS mean difference (95% CI)	XX.XX (XX.XX~XX.XX)	
P-value	XX.XX	

Note: Between-group differences were compared using Mixed-Effects Model for Repeated Measures (MMRM). The model included treatment group, visit, group-by-visit interaction, and baseline value as a covariate. Missing values were not imputed.

Table 4-12 Temporal comparison of between-group differences in LSMEANS of change in vitality score by MMRM (full analysis aet, FAS)

	Treatment group (N=XXX)	Placebo group (N=XXX)
Baseline		
N (NMissing)	XXX	XXX
Mean±SD	XX.XX±XX.XX	XX.XX±XX.XX
4 week		
N (NMissing)	XXX	XXX
Mean±SD	XX.XX±XX.XX	XX.XX±XX.XX
Change from baseline		
N (NMissing)	XXX	XXX
Mean±SD	XX.XX±XX.XX	XX.XX±XX.XX
LS mean (SE)	XX.XX (XX.XX)	XX.XX (XX.XX)
LS mean difference (95% CI)	XX.XX (XX.XX~XX.XX)	
P-value	XX.XX	
8 week		
N (NMissing)	XXX	XXX
Mean±SD	XX.XX±XX.XX	XX.XX±XX.XX
Change from baseline		
N (NMissing)	XXX	XXX
Mean±SD	XX.XX±XX.XX	XX.XX±XX.XX
LS mean (SE)	XX.XX (XX.XX)	XX.XX (XX.XX)
LS mean difference (95% CI)	XX.XX (XX.XX~XX.XX)	
P-value	XX.XX	
12 week		
N (NMissing)	XXX	XXX
Mean±SD	XX.XX±XX.XX	XX.XX±XX.XX
Change from baseline		
N (NMissing)	XXX	XXX
Mean±SD	XX.XX±XX.XX	XX.XX±XX.XX
LS mean (SE)	XX.XX (XX.XX)	XX.XX (XX.XX)
LS mean difference (95% CI)	XX.XX (XX.XX~XX.XX)	
P-value	XX.XX	

Note: Between-group differences were compared using Mixed-Effects Model for Repeated Measures (MMRM). The model included treatment group, visit, group-by-visit interaction, and baseline value as a covariate. Missing values were not imputed.

Table 4-13 Temporal comparison of between-group differences in LSMEANS of change in social functioning score by MMRM (full analysis set, FAS)

	Treatment group (N=XXX)	Placebo group (N=XXX)
Baseline		
N (NMissing)	XXX	XXX
Mean±SD	XX.XX±XX.XX	XX.XX±XX.XX
4 week		
N (NMissing)	XXX	XXX
Mean±SD	XX.XX±XX.XX	XX.XX±XX.XX
Change from baseline		
N (NMissing)	XXX	XXX
Mean±SD	XX.XX±XX.XX	XX.XX±XX.XX
LS mean (SE)	XX.XX (XX.XX)	XX.XX (XX.XX)
LS mean difference (95% CI)	XX.XX (XX.XX~XX.XX)	
P-value	XX.XX	
8 week		
N (NMissing)	XXX	XXX

	Treatment group (N=XXX)	Placebo group (N=XXX)
Mean±SD	XX.XX±XX.XX	XX.XX±XX.XX
Change from baseline		
N (NMissing)	XXX	XXX
Mean±SD	XX.XX±XX.XX	XX.XX±XX.XX
LS mean (SE)	XX.XX (XX.XX)	XX.XX (XX.XX)
LS mean difference (95% CI)	XX.XX (XX.XX~XX.XX)	
P-value	XX.XX	
12 week		
N (NMissing)	XXX	XXX
Mean±SD	XX.XX±XX.XX	XX.XX±XX.XX
Change from baseline		
N (NMissing)	XXX	XXX
Mean±SD	XX.XX±XX.XX	XX.XX±XX.XX
LS mean (SE)	XX.XX (XX.XX)	XX.XX (XX.XX)
LS mean difference (95% CI)	XX.XX (XX.XX~XX.XX)	
P-value	XX.XX	

Note: Between-group differences were compared using mixed-effects model for repeated measures (MMRM). The model included treatment group, visit, group-by-visit interaction, and baseline value as a covariate. Missing values were not imputed.

Table 4-14 Temporal comparison of between-group differences in LSMEANS of change in role-emotional score by MMRM (full analysis set, FAS)
Note: Between-group differences were compared using mixed-effects model for repeated measures (MMRM). The model included treatment group, visit, group-by-visit interaction, and baseline value as a covariate. Missing values were not imputed.

Table 4-15 Temporal comparison of between-group differences in LSMEANS of change in mental health score by MMRM (full analysis Set, FAS)

	Treatment group (N=XXX)	Placebo group (N=XXX)
Baseline		
N (NMissing)	XXX	XXX
Mean±SD	XX.XX±XX.XX	XX.XX±XX.XX
4 week		
N (NMissing)	XXX	XXX
Mean±SD	XX.XX±XX.XX	XX.XX±XX.XX
Change from baseline		
N (NMissing)	XXX	XXX
Mean±SD	XX.XX±XX.XX	XX.XX±XX.XX
LS mean (SE)	XX.XX (XX.XX)	XX.XX (XX.XX)
LS mean difference (95% CI)	XX.XX (XX.XX~XX.XX)	
P-value	XX.XX	
8 week		
N (NMissing)	XXX	XXX
Mean±SD	XX.XX±XX.XX	XX.XX±XX.XX
Change from baseline		
N (NMissing)	XXX	XXX
Mean±SD	XX.XX±XX.XX	XX.XX±XX.XX
LS mean (SE)	XX.XX (XX.XX)	XX.XX (XX.XX)
LS mean difference (95% CI)	XX.XX (XX.XX~XX.XX)	
P-value	XX.XX	
12 week		
N (NMissing)	XXX	XXX
Mean±SD	XX.XX±XX.XX	XX.XX±XX.XX
Change from baseline		

	Treatment group (N=XXX)	Placebo group (N=XXX)
N (NMissing)	XXX	XXX
Mean±SD	XX.XX±XX.XX	XX.XX±XX.XX
LS mean (SE)	XX.XX (XX.XX)	XX.XX (XX.XX)
LS mean difference (95% CI)	XX.XX (XX.XX~XX.XX)	
P-value	XX.XX	

Note: Between-group differences were compared using mixed-effects model for repeated measures (MMRM). The model included treatment group, visit, group-by-visit interaction, and baseline value as a covariate. Missing values were not imputed.

7.4.4 Anxiety and Depression Rating Scale Analysis

Table 4-16 Temporal comparison of between-group differences in LSMEANS of change in PHQ-9 score by MMRM (full analysis set, FAS)

	Treatment group (N=XXX)	Placebo group (N=XXX)
Baseline		
N (NMissing)	XXX	XXX
Mean±SD	XX.XX±XX.XX	XX.XX±XX.XX
4 week		
N (NMissing)	XXX	XXX
Mean±SD	XX.XX±XX.XX	XX.XX±XX.XX
Change from baseline		
N (NMissing)	XXX	XXX
Mean±SD	XX.XX±XX.XX	XX.XX±XX.XX
LS mean (SE)	XX.XX (XX.XX)	XX.XX (XX.XX)
LS mean difference (95% CI)	XX.XX (XX.XX~XX.XX)	
P-value	XX.XX	
8 week		
N (NMissing)	XXX	XXX
Mean±SD	XX.XX±XX.XX	XX.XX±XX.XX
Change from baseline		
N (NMissing)	XXX	XXX
Mean±SD	XX.XX±XX.XX	XX.XX±XX.XX
LS mean (SE)	XX.XX (XX.XX)	XX.XX (XX.XX)
LS mean difference (95% CI)	XX.XX (XX.XX~XX.XX)	
P-value	XX.XX	
12 week		
N (NMissing)	XXX	XXX
Mean±SD	XX.XX±XX.XX	XX.XX±XX.XX
Change from baseline		
N (NMissing)	XXX	XXX
Mean±SD	XX.XX±XX.XX	XX.XX±XX.XX
LS mean (SE)	XX.XX (XX.XX)	XX.XX (XX.XX)
LS mean difference (95% CI)	XX.XX (XX.XX~XX.XX)	
P-value	XX.XX	

Note: Between-group differences were compared using mixed-effects model for repeated measures (MMRM). The model included treatment group, visit, group-by-visit interaction, and baseline value as a covariate. Missing values were not imputed.

Table 4-17 Temporal comparison of between-group differences in LSMEANS of change in GAD-7 score by MMRM (full analysis set, FAS)

	Treatment group (N=XXX)	Placebo group (N=XXX)
Baseline		
N (NMissing)	XXX	XXX
Mean±SD	XX.XX±XX.XX	XX.XX±XX.XX
4 week		

	Treatment group (N=XXX)	Placebo group (N=XXX)
N (NMissing)	XXX	XXX
Mean±SD	XX.XX±XX.XX	XX.XX±XX.XX
Change from baseline		
N (NMissing)	XXX	XXX
Mean±SD	XX.XX±XX.XX	XX.XX±XX.XX
LS mean (SE)	XX.XX (XX.XX)	XX.XX (XX.XX)
LS mean difference (95% CI)	XX.XX (XX.XX~XX.XX)	
P-value	XX.XX	
8 week		
N (NMissing)	XXX	XXX
Mean±SD	XX.XX±XX.XX	XX.XX±XX.XX
Change from baseline		
N (NMissing)	XXX	XXX
Mean±SD	XX.XX±XX.XX	XX.XX±XX.XX
LS mean (SE)	XX.XX (XX.XX)	XX.XX (XX.XX)
LS mean difference (95% CI)	XX.XX (XX.XX~XX.XX)	
P-value	XX.XX	
12 week		
N (NMissing)	XXX	XXX
Mean±SD	XX.XX±XX.XX	XX.XX±XX.XX
Change from baseline		
N (NMissing)	XXX	XXX
Mean±SD	XX.XX±XX.XX	XX.XX±XX.XX
LS mean (SE)	XX.XX (XX.XX)	XX.XX (XX.XX)
LS mean difference (95% CI)	XX.XX (XX.XX~XX.XX)	
P-value	XX.XX	

Note: Between-group differences were compared using mixed-effects model for repeated measures (MMRM). The model included treatment group, visit, group-by-visit interaction, and baseline value as a covariate. Missing values were not imputed.

Table 4-18 Temporal comparison of between-group differences in LSMEANS of change in Self-Rating Depression Scale (SDS) score by MMRM (full analysis set, FAS)

	Treatment group (N=XXX)	Placebo group (N=XXX)
Baseline		
N (NMissing)	XXX	XXX
Mean±SD	XX.XX±XX.XX	XX.XX±XX.XX
4 week		
N (NMissing)	XXX	XXX
Mean±SD	XX.XX±XX.XX	XX.XX±XX.XX
Change from baseline		
N (NMissing)	XXX	XXX
Mean±SD	XX.XX±XX.XX	XX.XX±XX.XX
LS mean (SE)	XX.XX (XX.XX)	XX.XX (XX.XX)
LS mean difference (95% CI)	XX.XX (XX.XX~XX.XX)	
P-value	XX.XX	
8 week		
N (NMissing)	XXX	XXX
Mean±SD	XX.XX±XX.XX	XX.XX±XX.XX
Change from baseline		
N (NMissing)	XXX	XXX
Mean±SD	XX.XX±XX.XX	XX.XX±XX.XX
LS mean (SE)	XX.XX (XX.XX)	XX.XX (XX.XX)
LS mean difference (95% CI)	XX.XX (XX.XX~XX.XX)	
P-value	XX.XX	

	Treatment group (N=XXX)	Placebo group (N=XXX)
12 week		
N (NMissing)	XXX	XXX
Mean±SD	XX.XX±XX.XX	XX.XX±XX.XX
Change from baseline		
N (NMissing)	XXX	XXX
Mean±SD	XX.XX±XX.XX	XX.XX±XX.XX
LS mean (SE)	XX.XX (XX.XX)	XX.XX (XX.XX)
LS mean difference (95% CI)	XX.XX (XX.XX~XX.XX)	
P-value	XX.XX	

Note: Between-group differences were compared using mixed-effects model for repeated measures (MMRM). The model included treatment group, visit, group-by-visit interaction, and baseline value as a covariate. Missing values were not imputed.

Table 4-19 Temporal comparison of between-group differences in LSMEANS of change in Self-Rating Anxiety Scale (SAS) score by MMRM (full analysis set, FAS)

	Treatment group (N=XXX)	Placebo group (N=XXX)
Baseline		
N (NMissing)	XXX	XXX
Mean±SD	XX.XX±XX.XX	XX.XX±XX.XX
4 week		
N (NMissing)	XXX	XXX
Mean±SD	XX.XX±XX.XX	XX.XX±XX.XX
Change from baseline		
N (NMissing)	XXX	XXX
Mean±SD	XX.XX±XX.XX	XX.XX±XX.XX
LS mean (SE)	XX.XX (XX.XX)	XX.XX (XX.XX)
LS mean difference (95% CI)	XX.XX (XX.XX~XX.XX)	
P-value	XX.XX	
8 week		
N (NMissing)	XXX	XXX
Mean±SD	XX.XX±XX.XX	XX.XX±XX.XX
Change from baseline		
N (NMissing)	XXX	XXX
Mean±SD	XX.XX±XX.XX	XX.XX±XX.XX
LS mean (SE)	XX.XX (XX.XX)	XX.XX (XX.XX)
LS mean difference (95% CI)	XX.XX (XX.XX~XX.XX)	
P-value	XX.XX	
12 week		
N (NMissing)	XXX	XXX
Mean±SD	XX.XX±XX.XX	XX.XX±XX.XX
Change from baseline		
N (NMissing)	XXX	XXX
Mean±SD	XX.XX±XX.XX	XX.XX±XX.XX
LS mean (SE)	XX.XX (XX.XX)	XX.XX (XX.XX)
LS mean difference (95% CI)	XX.XX (XX.XX~XX.XX)	
P-value	XX.XX	

Note: Between-group differences were compared using mixed-effects model for repeated measures (MMRM). The model included treatment group, visit, group-by-visit interaction, and baseline value as a covariate. Missing values were not imputed.

7.4.5 Sex Hormones

Table 4-20 Temporal analysis of measured values of estradiol (pg/ml) (full analysis set, FAS)

Visit	Treatment group (N=XXX)	Placebo group (N=XXX)
Baseline		

Visit	Treatment group (N=XXX)	Placebo group (N=XXX)
N (NMissing)	XXX	XXX
Median (IQR)	XX.XX (XX.X, XX.X)	XX.XX (XX.X, XX.X)
8 week		
N (NMissing)	XXX	XXX
Median (IQR)	XX.XX (XX.X, XX.X)	XX.XX (XX.X, XX.X)
Change from baseline		
N (NMissing)	XXX	XXX
Median (IQR)	XX.XX (XX.X, XX.X)	XX.XX (XX.X, XX.X)
Between-group difference		
Median (IQR)	XX.XX (XX.X, XX.X)	
P-value	XX.XX	

Note: Between-group differences were compared using the Wilcoxon test.

Table 4-21 Temporal analysis of measured values of progesterone (nmol/L) (full analysis set, FAS)

Visit	Treatment group (N=XXX)	Placebo group (N=XXX)
Baseline		
N (NMissing)	XXX	XXX
Median (IQR)	XX.XX (XX.X, XX.X)	XX.XX (XX.X, XX.X)
8 week		
N (NMissing)	XXX	XXX
Median (IQR)	XX.XX (XX.X, XX.X)	XX.XX (XX.X, XX.X)
Change from baseline		
N (NMissing)	XXX	XXX
Median (IQR)	XX.XX (XX.X, XX.X)	XX.XX (XX.X, XX.X)
Between-group difference		
Median (IQR)	XX.XX (XX.X, XX.X)	
P-value	XX.XX	

Note: Between-group differences were compared using the Wilcoxon test.

Table 4-22 Temporal analysis of measured values of prolactin (ng/ml) (full analysis set, FAS)

Visit	Treatment group (N=XXX)	Placebo group (N=XXX)
Baseline		
N (NMissing)	XXX	XXX
Median (IQR)	XX.XX (XX.X, XX.X)	XX.XX (XX.X, XX.X)
8 week		
N (NMissing)	XXX	XXX
Median (IQR)	XX.XX (XX.X, XX.X)	XX.XX (XX.X, XX.X)
Change from baseline		
N (NMissing)	XXX	XXX
Median (IQR)	XX.XX (XX.X, XX.X)	XX.XX (XX.X, XX.X)
Between-group difference		
Median (IQR)	XX.XX (XX.X, XX.X)	
P-value	XX.XX	

Note: Between-group differences were compared using the Wilcoxon test.

7.4.6 Cytokines

Table 4-23 Temporal analysis of measured values of tumor necrosis factor-α (pm/ml) (full analysis set, FAS)

Visit	Treatment group (N=XXX)	Placebo group (N=XXX)
Baseline		
N (NMissing)	XXX	XXX
Median (IQR)	XX.XX (XX.X, XX.X)	XX.XX (XX.X, XX.X)
8 week		
N (NMissing)	XXX	XXX
Median (IQR)	XX.XX (XX.X, XX.X)	XX.XX (XX.X, XX.X)

Visit	Treatment group (N=XXX)	Placebo group (N=XXX)
Change from baseline		
N (NMissing)	XXX	XXX
Median (IQR)	XX.XX (XX.X, XX.X)	XX.XX (XX.X, XX.X)
Between-group difference		
Median (IQR)	XX.XX (XX.X, XX.X)	
P-value	XX.XX	

Note: Between-group differences were compared using the Wilcoxon test.

Table 4-24 Temporal analysis of measured values of interleukin-6 (pm/ml) (full analysis set, FAS)

Visit	Treatment group (N=XXX)	Placebo group (N=XXX)
Baseline		
N (NMissing)	XXX	XXX
Median (IQR)	XX.XX (XX.X, XX.X)	XX.XX (XX.X, XX.X)
8 week		
N (NMissing)	XXX	XXX
Median (IQR)	XX.XX (XX.X, XX.X)	XX.XX (XX.X, XX.X)
Change from baseline		
N (NMissing)	XXX	XXX
Median (IQR)	XX.XX (XX.X, XX.X)	XX.XX (XX.X, XX.X)
Between-group difference		
Median (IQR)	XX.XX (XX.X, XX.X)	
P-value	XX.XX	

Note: Between-group differences were compared using the Wilcoxon test.

7.5 Safety Analysis

7.5.1 Adverse Event Analysis

Table 5-1 Analysis of incidence of adverse events (Safety set, SS)

Indicator	Treatment group (N=XXX)	Placebo group (N=XXX)	P-value
N	XXX	XXX	
Adverse events *n* (%)	XX (XX.X)	XX (XX.X)	X.XXX
Adverse reactions *n* (%)	XX (XX.X)	XX (XX.X)	X.XXX
Serious adverse events *n* (%)	XX (XX.X)	XX (XX.X)	X.XXX
Serious adverse reactions *n* (%)	XX (XX.X)	XX (XX.X)	X.XXX
Adverse events leading to withdrawal *n* (%)	XX (XX.X)	XX (XX.X)	X.XXX
Adverse reactions leading to withdrawal *n* (%)	XX (XX.X)	XX (XX.X)	X.XXX
Adverse events leading to death *n* (%)	XX (XX.X)	XX (XX.X)	X.XXX

Note: Adverse events with a relationship to the study drug classified as “definitely related”, “probably related”, or “possibly related” are considered drug-related TEAEs (i.e., ADRs).

Table 5-2 Systemic analysis of adverse events (safety set, SS)
Table 5-3 Systemic analysis of adverse reactions (safety set, SS)
Table 5-4 Systemic analysis of serious adverse events (safety set, SS)
Table 5-5 Systemic analysis of serious adverse reactions (safety set, SS)
Table 5-6 Systemic analysis of adverse events leading to withdrawal from the study (safety set, SS)

Table 5-7 Systemic analysis of adverse reactions leading to withdrawal from the study (safety set, SS)

SOC	Treatment group (N=XXX)		Placebo group (N=XXX)	
PT	Number of cases	Number of occurrences	Number of cases	Number of occurrences
N	XXX		XXX	
Summary n (%)	XXX (XX.X)	XXX	XXX (XX.X)	XXX
SOC1 n (%)	XXX (XX.X)	XXX	XXX (XX.X)	XXX
PT1 n (%)	XXX (XX.X)	XXX	XXX (XX.X)	XXX
PT2 n (%)	XXX (XX.X)	XXX	XXX (XX.X)	XXX
…				
SOC2	XXX (XX.X)	XXX	XXX (XX.X)	XXX
PT1 n (%)	XXX (XX.X)	XXX	XXX (XX.X)	XXX
PT2 n (%)	XXX (XX.X)	XXX	XXX (XX.X)	XXX
…				
SOC3	XXX (XX.X)	XXX	XXX (XX.X)	XXX
PT1 n (%)	XXX (XX.X)	XXX	XXX (XX.X)	XXX
PT2 n (%)	XXX (XX.X)	XXX	XXX (XX.X)	XXX
…				
